# Phylogenomic analysis of Syngnathidae reveals novel relationships, origins of endemic diversity and variable diversification rates

**DOI:** 10.1186/s12915-022-01271-w

**Published:** 2022-03-27

**Authors:** Josefin Stiller, Graham Short, Healy Hamilton, Norah Saarman, Sarah Longo, Peter Wainwright, Greg W. Rouse, W. Brian Simison

**Affiliations:** 1 Scripps Institution of Oceanography, University of California San Diego, La Jolla, USA; 2 Centre for Biodiversity Genomics, University of Copenhagen, 2100 Copenhagen, Denmark; 3 Ichthyology, Australian Museum, Sydney, Australia; 4 Ichthyology, California Academy of Sciences, San Francisco, USA; 5 Ichthyology, Burke Museum of Natural History and Culture, Seattle, USA; 6 NatureServe, Arlington, USA; 7 Department of Biology and Ecology Center, Utah State University, Logan, Utah USA; 8 Department of Biological Science, Towson University, Towson, MD 21252 USA; 9 Department of Evolution & Ecology, University of California, Davis, USA; 10 Center for Comparative Genomics, California Academy of Sciences, San Francisco, USA

**Keywords:** Phylogeny, Diversification, Biogeography, Syngnathidae, Seahorses, UCEs

## Abstract

**Background:**

Seahorses, seadragons, pygmy pipehorses, and pipefishes (Syngnathidae, Syngnathiformes) are among the most recognizable groups of fishes because of their derived morphology, unusual life history, and worldwide distribution. Despite previous phylogenetic studies and recent new species descriptions of syngnathids, the evolutionary relationships among several major groups within this family remain unresolved.

**Results:**

Here, we provide a reconstruction of syngnathid phylogeny based on genome-wide sampling of 1314 ultraconserved elements (UCEs) and expanded taxon sampling to assess the current taxonomy and as a basis for macroevolutionary insights. We sequenced a total of 244 new specimens across 117 species and combined with published UCE data for a total of 183 species of Syngnathidae, about 62% of the described species diversity, to compile the most data-rich phylogeny to date. We estimated divergence times using 14 syngnathiform fossils, including nine fossils with newly proposed phylogenetic affinities, to better characterize current and historical biogeographical patterns, and to reconstruct diversification through time. We present a phylogenetic hypothesis that is well-supported and provides several notable insights into syngnathid evolution. We found nine non-monophyletic genera, evidence for seven cryptic species, five potentially invalid synonyms, and identified a novel sister group to the seahorses, the Indo-Pacific pipefishes *Halicampus macrorhynchus* and *H. punctatus*. In addition, the morphologically distinct southwest Pacific seahorse *Hippocampus jugumus* was recovered as the sister to all other non-pygmy seahorses. As found in many other groups, a high proportion of syngnathid lineages appear to have originated in the Central Indo-Pacific and subsequently dispersed to adjoining regions. Conversely, we also found an unusually high subsequent return of lineages from southern Australasia to the Central Indo-Pacific. Diversification rates rose abruptly during the Middle Miocene Climate Transition and peaked after the closure of the Tethys Sea.

**Conclusions:**

Our results reveal a previously underappreciated diversity of syngnathid lineages. The observed biogeographic patterns suggest a significant role of the southern Australasian region as a source and sink of lineages. Shifts in diversification rates imply possible links to declining global temperatures, the separation of the Atlantic and Pacific faunas, and the environmental changes associated with these events.

**Supplementary Information:**

The online version contains supplementary material available at 10.1186/s12915-022-01271-w.

## Background

Syngnathidae—pipefishes, seahorses, pygmy pipehorses, and seadragons—are among the most easily recognized fishes that fascinate aquarium visitors, SCUBA divers, and scientists alike. There are numerous characteristics that make syngnathids noteworthy, including their elongated snouts, external skeletal armor, prehensile tail, crypsis, and male brooding. Syngnathids are models for studying questions of sexual selection and sex roles [1–3], as well as the evolution of flexible armor [4, 5], and genomic evolution [6, 7].

The placement of Syngnathidae within Syngnathiformes, a taxonomically, morphologically, and ecologically diverse group (comprising the families Pegasidae, Callionymidae, Dactylopteridae, Draconettidae, Mullidae, Centriscidae, Fistulariidae, Aulostomidae, Solenostomidae, and Syngnathidae) is now well established [8–11]. Syngnathids share ovoviviparity with their sister group, the ghost pipefishes (Solenostomidae), though the female broods the eggs in modified pelvic fins in the latter clade. The 296 species of Syngnathidae (Additional file 1: Table S1) [12–42] comprise various pipefishes and other iconic groups such as seadragons, pygmy pipehorses, and seahorses [43, 44]. Previous phylogenetic studies have found that morphologically defined groups were often not reflective of evolutionary history. Examples include three genera that were recovered as paraphyletic [44], syngnathids referred to as “seadragons” that were shown to consist of two phylogentically distinct groups with convergently evolved leaf-like appendages [45], and morphologically similar members of “pygmy pipehorses,” previously retained in the same subgenus [43], which were found to be two distantly related lineages [44].

In recent years, significant advances have been made in understanding the evolutionary relationships among Syngnathidae. The taxonomically best sampled phylogeny included 91 species of syngnathids [44]; however, only a few mitochondrial and nuclear genes were analyzed, resulting in low resolution of some principal branches. Phylogenomic studies using genome-wide ultraconserved elements (UCEs) across Syngnathiformes resulted in largely robust relationships but were limited in their taxon sampling of Syngnathidae [9, 11]. A total of 57 syngnathid species were sampled by [9], to which 14 species were added [11] leaving ca. 3/4 of the described species diversity unsampled. This undersampling also concerns extinct species of Syngnathidae and Syngnathiformes, for which a relatively good fossil record exists particularly from the former Tethys Sea because their outer bony plates fossilize well [46–48]. However, prior attempts to estimate divergence times focused primarily on seahorse fossils from the Miocene for time calibration [11, 49], omitting a substantial number of other fossil taxa.

The evolutionary history of seahorses (*Hippocampus*) has garnered notable attention [49, 50], but key phylogenetic questions remain unresolved, including the placement of some species, the identity of the closest living relative of seahorses, and the time frame of their divergence. *Hippocampus* comprises 42 species [51] (Additional file 1: Table S1) with pygmy seahorses and non-pygmy seahorses grouped into two phylogenetically distinct lineages [44, 52]. Among previously unsampled seahorse species, *H. jugumus* is of particular interest because it differs from its congeners by atypical characteristics (such as slender body, high meristic counts, double spines, and a continuous cleithrum [53]); therefore, elucidating its phylogenetic placement is important to understand character evolution within *Hippocampus*. The sister group to seahorses has remained unresolved; for example, the pygmy pipehorses were long suggested as a candidate due to their shared morphological traits, such as the angled head and prehensile tail [54], which was supported in one multigene analysis [52]. However, a more thorough taxon sampling revealed that pygmy pipehorses were not monophyletic and neither lineage was closely related to the seahorses; thereby, no strongly supported proposal as yet has been established [44]. Lastly, the most recent attempts to date divergences within seahorses have resulted in age estimates for the non-pygmy seahorses that are 10 million years apart because of different interpretations of the same fossil [11, 49]. Improved phylogenetic resolution with robust estimates of divergence times is needed to help resolve questions about biogeographic and morphological evolution of this iconic group.

Another uncertainty revolves around the geographic origins of syngnathid diversity. Syngnathids are found in every ocean basin except the polar regions and a number of lineages occur in brackish or freshwater [43]. As with many shallow marine groups, the greatest diversity of Syngnathidae is found in the tropical Indo-Pacific, which is home to 226 species. A significant proportion of diversity is also found in temperate southern Australia and New Zealand (south of -28° of latitude); of a total 43 species, 90% are endemic to the region [43, 44]. The southern coast also harbors significant endemic diversity of other marine groups [55], whereas the tropical regions of Australia have a stronger overlap in species distribution with the Central Indo-Pacific. The temperate region includes over 15,000 km of coastline, with some of the world’s most extensive seagrass meadows and productive rocky reefs with large kelp forests [56]. A previous study inferred multiple colonization events for Syngnathidae from the Central Indo-Pacific to the temperate Australasian region [11], but it did not include several key lineages of the endemic fauna (including temperate members of *Festucalex*, *Heraldia*, *Leptonotus*, *Lissocampus*, *Maroubra*, *Mitotichthys*, *Stipecampus*, *Urocampus*). By nature of their life history (brooding, small home ranges, low fecundity, distinct breeding periods, poor juvenile and adult dispersal), syngnathids are excellent models to determine the biogeographic origins of this remarkable diversity of southern Australian endemic fishes.

Here, we investigated the global diversification history of Syngnathidae with the most comprehensive locus and taxon sampling to date, incorporating sequences from 1314 nuclear UCEs [57, 58] for 303 specimens among 183 species of Syngnathidae, representing ~62% of the extant diversity. We integrate this sequence data with a previously published UCE dataset of Syngnathiformes [9] and analyze it with an expanded compilation of 14 syngnathiform fossils to generate a robust time calibration. We then quantify the geographic patterns and diversification rates through time leading to present-day diversity. Our results resolve previously intractable relationships, reveal new phylogenetic patterns, and provide insights on the dynamic evolutionary history of this unusual group of marine fishes.

## Results

### Sampling and data characteristics

We analyzed UCE data for a total 361 individuals of Syngnathiformes (including one Scombriformes as the outgroup) in 238 species, including 248 newly sequenced samples and 113 published samples [9] (Additional file 2). The included samples were obtained from 28 museum collections and public aquaria. Our dataset doubles the number of syngnathid species represented in previous studies [9, 11, 44], representing a total of 183 species (54/58 genera) and ~62% of the described species diversity.

Newly sequenced samples had, on average, 1.8 million paired reads (range 30,396–12,940,432) after quality trimming and read de-duplication and each sample assembled, on average, 949 UCE loci (range 318–1091, Additional file 2). The concatenated matrices had a high number of loci and sites, with the main matrix (75% completeness) for the 361 taxa having 934 loci with 236,733 bp and 110,393 parsimony-informative sites (metrics for other matrices in Additional file 1: Table S4). Coalescent-based analyses were performed on gene trees from 1309 loci with a mean locus length of 230 bp (Additional file 1: Table S4).

### Phylogenetic relationships of Syngnathidae

**Fig. 1:**
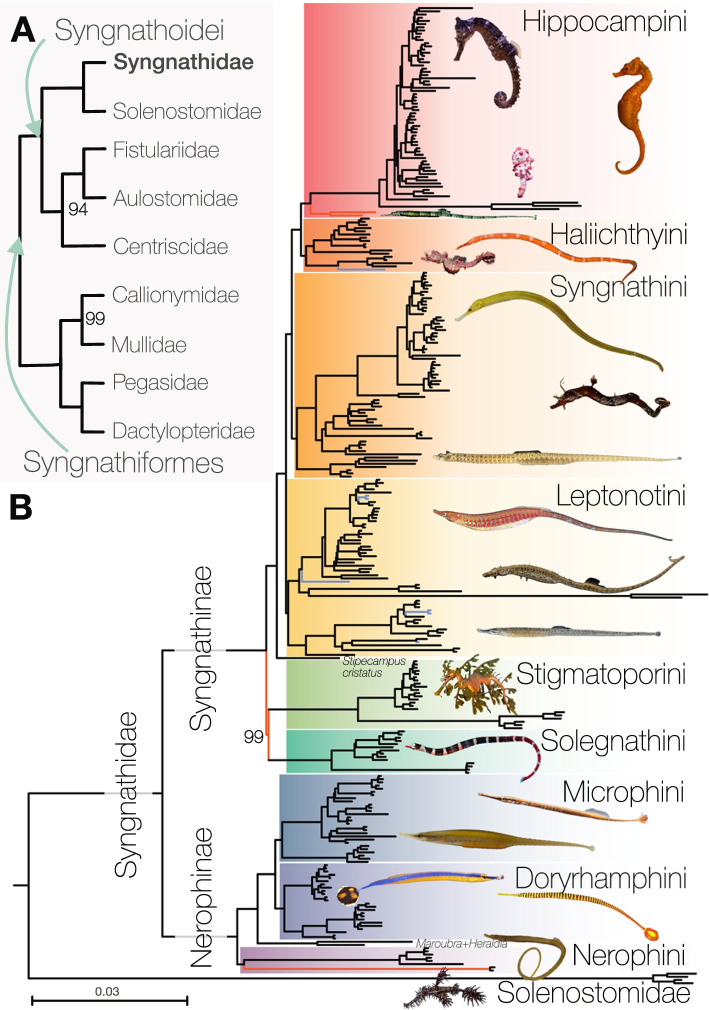
Overview of phylogenetic relationships of Syngnathiformes and Syngnathidae. **A** Overview of the topology among the syngnathiform families. **B** Relationships among Syngnathidae and Solenostomidae. The nine tribes of Syngnathidae are highlighted in color and are shown in detail in Figs. 2 and 3. Highlighted branches indicate newly sampled genera (blue) and main different relationships to other studies (red), as discussed in the text. The tree topology is based on the multi-individual dataset (361 individuals, 238 species) based on the 75% complete matrix (934 loci, 236,744 bp) analyzed in IQ-TREE2. Node labels are only annotated for bootstrap support <100 between families and tribes; detailed relationships are shown in Figs. 2 and 3

The maximum likelihood and coalescent-based trees of 361 taxa had overall high support (Fig. 1, Additional file 1: Figs. S1-S6). As previously found, relationships between Syngnathiformes differed depending on the analytical framework [9, 11]: In concatenation analyses, Dactylopteridae, Pegasidae, Mullidae, and Callionymidae formed a clade (Fig. 1A), while coalescent-based analyses placed those taxa as a grade (Additional file 1: Fig. S1). The remainder of the syngnathiform lineages were well supported, with Centriscidae, Aulostomidae, and Fistulariidae being the sister group to Solenostomidae and Syngnathidae (=Syngnathoidei) [9, 11]. These results differ from analyses based on protein-coding genes, where Fistulariidae, separate from Centriscidae, were the sister group to Solenostomidae and Syngnathidae [10]. Solenostomidae were unambiguously supported as the sister group to Syngnathidae [9, 11], and within Syngnathidae, we also found the primary split into trunk-brooders (Nerophinae) and tail brooders (Syngnathinae) (Fig. 1B) [9, 44, 59].

Our analyses provide fine-scale insights into the primary clades of Syngnathidae, nine of which we assign here with the zoological nomenclatural rank of tribes (Fig. 1B, fully labeled tree Additional file 1: Fig. S7). We use available names where possible and name three new tribes (type taxa and diagnoses in Additional file 1: Systematics).

**Fig. 2:**
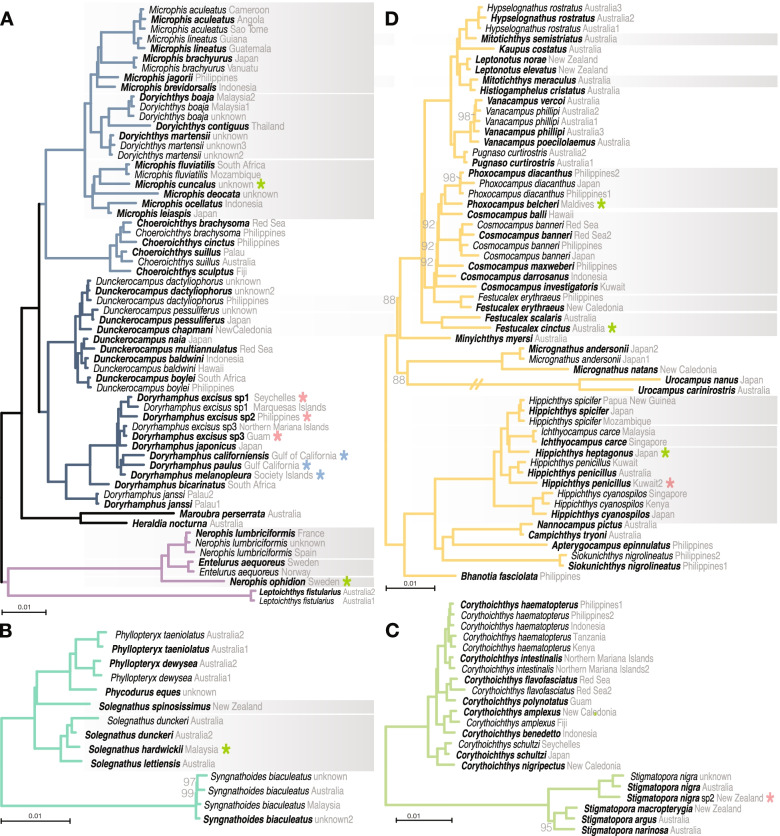
Detailed phylogenetic relationships within tribes of Nerophinae and Syngnathinae. Node labels are only annotated for bootstrap support <100. Terminals in bold are the specimens used to represent the species for the time calibration in Fig. 5. Stars annotate previously synonymized species (blue), potentially undescribed species (red), and type species for non-monophyletic genera if sampled (green). Grey boxes annotate non-monophyletic genera. Enlarged phylogenetic relationships are shown for **A** Nerophinae including Nerophini, Doryramphini and Microphini, **B** Solegnathini, **C** Stigmatoporini, and **D **Leptonotini

Nerophinae was found to be composed of an unnamed clade of *Maroubra* and *Heraldia nocturna* and three tribes; *Nerophis*, *Entelurus* and *Leptoichthys fistularius* (Nerophini); flagtail pipefishes (Doryrhamphini); a clade including some of the freshwater pipefishes (Microphini) (Fig. 2A). The most notable difference from previous phylogenetic hypotheses is in the placement of *Leptoichthys*, before placed as the sister to all Nerophinae [44], whereas here highly supported as part of Nerophini (bootstrap=100, posterior probability=0.98, Fig. 2A, Additional file 1: Fig. S1).

**Fig. 3:**
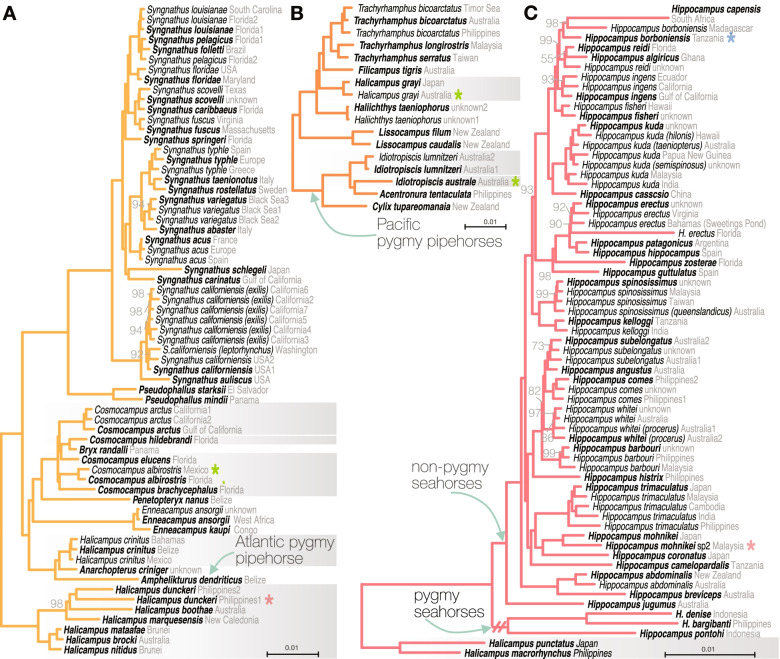
Detailed phylogenetic relationships within tribes of Syngnathinae. Node labels are only annotated for bootstrap support <100. Terminals in bold are the specimens used to represent the species for the time calibration in Fig. 5. Stars annotate previously synonymized species (blue), potentially undescribed species (red), and type species for non-monophyletic genera if sampled (green). Grey boxes annotate non-monophyletic genera. Enlarged phylogenetic relationships are shown for **A** Syngnathini, **B** Haliichthyini, and **C** Hippocampini

Syngnathinae were found to contain six tribes; seadragons and relatives (Solegnathini, Fig. 2B); *Stigmatopora* and *Corythoichthys* (Stigmatoporini, Fig. 2C); a group of diverse pipefishes (Leptonotini, Fig. 2D); the Atlantic pygmy pipehorse, the speciose *Syngnathus* and other pipefishes (Syngnathini, Fig. 3A); Pacific pygmy pipehorses and other pipefishes (Haliichthyini, Fig. 3B); seahorses, *Halicampus macrorhynchus* and *H. punctatus* (Hippocampini, Fig. 3C). The monotypic *Stipecampus cristatus*, the sister group to Leptonotini, Syngnathini, Haliichthyini and Hippocampini, was not placed into any higher taxon as such a name is redundant. The placement of Solegnathini and Stigmatoporini as sister groups was well supported in concatenation (bootstrap support=99, Fig. 1B) but only poorly supported in coalescent-based trees (posterior probability=54; Additional file 1: Fig. S1). The relationship agrees with previous studies based on UCEs [9, 11], while multigene analyses found Stigmatoporini to branch off first as the sister to all other Syngnathinae [44, 46]. All other relationships between tribes were resolved with highest bootstrap support.

Within Hippocampini, we found a new sister group to the seahorses, a clade of Indo-Pacific pipefish species *Halicampus macrorhynchus* and *H. punctatus* (Fig. 3C). This relationship was fully supported across all analyses. Within *Hippocampus*, pygmy seahorses formed a long branch that was the sister group to the speciose non-pygmy seahorses, aligning with previous studies [44, 52]. The morphologically distinct *H. jugumus*, included for the first time in a molecular study, was recovered as the sister to all other non-pygmy seahorses.

Of the genera that had not been included in previous molecular studies (blue branches in Fig. 1B), most were found to be within Leptonotini (Fig. 2D). *Campichthys tryoni* was supported as the sister group to *Nannocampus* as part of a group of pipefishes with reduced fins (*Apterygocampus*, *Siokunichthys*). *Ichthyocampus* was found to be part of *Hippichthys* (see below)*. Minyichthys myersi* was supported as the sister species to a large group of Indo-Pacific pipefishes. *Leptonotus elevatus* and *L. norae*, both endemic to New Zealand, were recovered within a large clade of southern Australasian species. In Haliichthyini, the recently described genus *Cylix* was found to be the sister group to other Indo-Pacific pygmy pipehorses (Fig. 3C).

### Taxonomic findings

Our phylogenomic hypothesis helped to clarify taxonomic uncertainties for several taxa, including five species that are currently treated as synonyms of other species (blue stars in Figs. 2 and 3; Additional file 1: Taxonomy). These concern the flagtail pipefishes *Doryrhamphus melanopleura*, *D. paulus* and *D. californiensis*, the seahorse *H. borboniensis*, and the ghost pipefish *Solenostomus paegnius*. For nine species, we also record expansions to the known geographic ranges or confirm unusually wide ranges.

Seven potentially undescribed species are noted based on branch length differences to putative conspecifics (red stars in Figs. 2 and 3, Additional file 1: Taxonomy). These include *Hippichthys penicillus* and *Halicampus dunckeri*, both of which showed long branches separating individuals from close-by localities. We confirm at least three unnamed species in *D. excisus* [60] and one in *Stigmatopora nigra* [61]. Our findings also support a potential separate species in the Japanese seahorse *H. mohnikei* [51], which we confirmed with COI barcodes obtained from the sequence data. Our Malaysian specimen has an identical COI sequence to specimens from India. This Indian occurrence was previously interpreted as evidence of a range expansion of *H. mohnikei* [62], but with a minimum divergence of 7.8% from nominal *H. mohnikei*, the interpretation as a separate species may be more justified.

Nine genera were identified as non-monophyletic (grey boxes in Figs. 2 and 3). Four of these were previously found (*Cosmocampus* [11, 44], *Halicampus* [44], *Microphis* [9, 11], *Solegnathus* [11]), to which we added confidence in their delimitation by including additional species and particularly specimens for the type species (green stars in Figs. 2 and 3). New evidence is presented for five other non-monophyletic genera (*Festucalex*, *Hippichthys*, *Mitotichthys*, *Nerophis*, *Idiotropiscis*). The northeastern Atlantic genus *Nerophis* contained the monotypic *Entelurus aequoreus* (Fig. 2A). Within Leptonotini, species of *Festucalex* fell into two groups, the euryhaline *Hippichthys* was found to contain the monotypic freshwater pipefish *Ichthyocampus carce*, and the two included species of *Mitotichthys* occurred in two clades (Fig. 2D). The sister relationship between *Idiotropiscis australe* and *Acentronura tentaculata* to the exclusion of *I. lumnitzeri* (Fig. 3B) is surprising because it contradicts morphological characters [43, 63]. Further study including the type species *A. gracilissima* is needed to justify potential taxonomic changes in the Pacific pygmy pipehorses. Altogether, these findings show that large portions of syngnathid organization require revision.

### Diversity and endemism

**Fig. 4:**
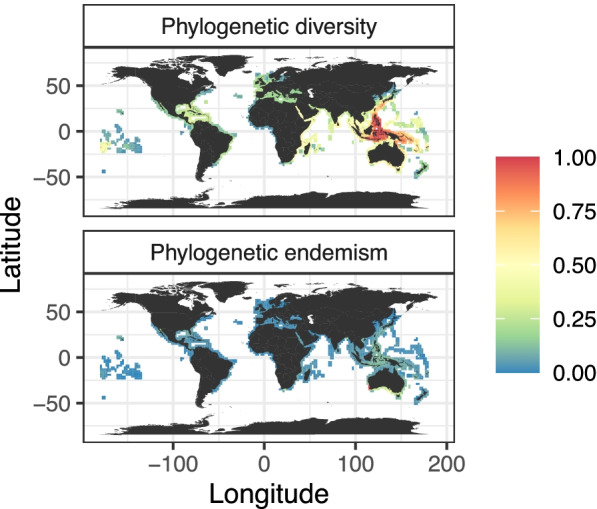
Global diversity of Syngnathidae and Solenostomidae. Spatial distribution of phylogenetic diversity (i.e. the sum of all branches) and phylogenetic endemism (i.e. spatially restricted phylogenetic diversity) calculated on a geographic grid. Values are scaled to range between 0 and 1

The Central Indo-Pacific was the richest biogeographic region to be represented by our sampling of Syngnathidae and Solenostomidae (93 species), of which less than half were endemic to the region because of extensive sharing mostly with other tropical areas (37 species, 40%). Species occurring in temperate Australia on the other hand (41 species) were largely endemic to this region (36 species, 88%). These distributions were also reflected in the spatial patterns of diversity: While phylogenetic diversity (i.e., the sum of all branches) was highest in the Central Indo-Pacific (Fig. 4), phylogenetic endemism (i.e., the phylogenetic diversity that is spatially restricted) was globally low but notably heightened in temperate Australia (Fig. 4).

### Timescale of syngnathid diversification

After review of the fossils described for Syngnathidae and other Syngnathiformes, we applied 14 calibrations in a Bayesian node-dating framework (Additional file 1: Fossil Justifications, Fig. S8). Based on shared similarities to extant taxa, we propose new placements for nine fossil taxa. Deriving new positions was particularly successful within Nerophinae, where we identified morphological traits that appear the be synapomorphies for clades. For example, Microphini, Doryrhamphini, *Maroubra*, and *Heraldia* all share spiny ridges on their body rings (Additional file 1: Table S2). We note that two of the oldest node calibrations used previously [11] are predated by older fossils from other localities: The oldest stem Pegasidae †*Ramphosus rosenkrantzi* from the Danish Fur Formation (ca. 54 Ma) is older than †*R. rastrum* from Monte Bolca in Italy (ca. 48.5 Ma), and the stem Fistulariidae †*Urosphenopsis sagitta* of the Danatinsk Formation of Turkmenistan (ca. 54.17 Ma) predates †*Urosphen dubius* from Monte Bolca.

The resulting age estimates were largely robust when different subsets of the fossil calibrations were used: When excluding the oldest fossil constraint on stem Syngnathoidei, the nodes were on average slightly older (mean = 0.63, median = 0.28, Additional file 1: Fig. S9), and removing the seven syngnathid calibrations resulted in somewhat younger node estimates but no drastic changes (mean = −1.75, median = −1.21, Additional file 1: Fig. S10).

**Fig. 5:**
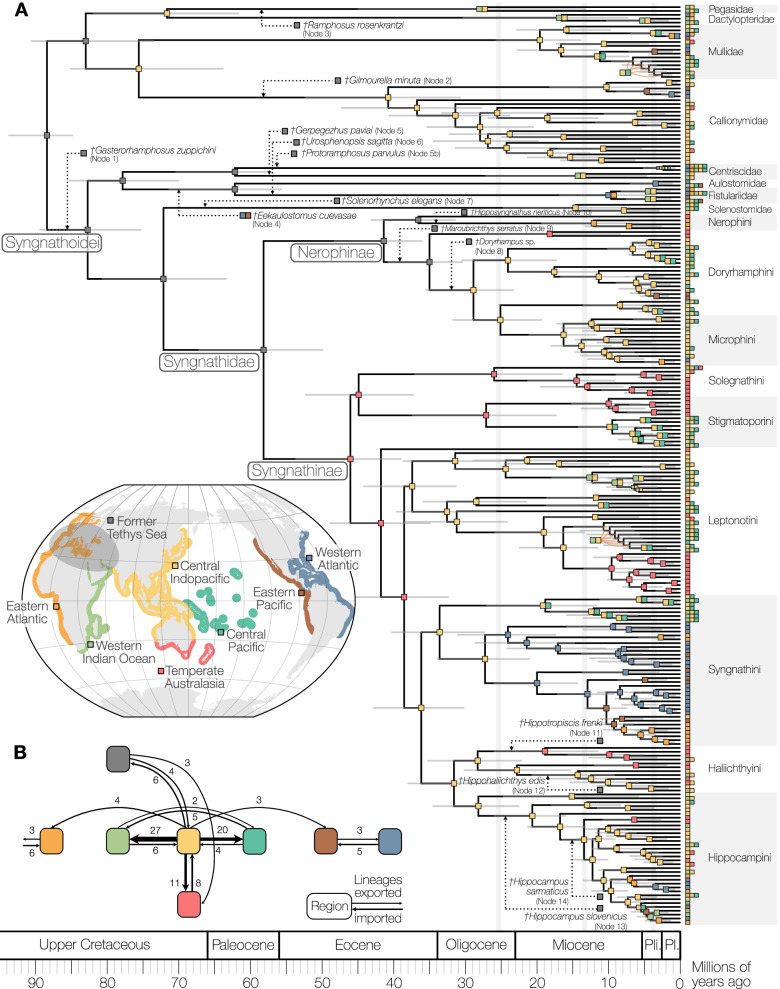
Time frame of diversification and biogeographic evolution of Syngnathiformes. **A** Time-calibrated tree of Syngnathiformes and biogeographic history based on the dataset with 238 species. Fossil calibration was performed in BEAST2 using 14 syngnathiform fossils on a partitioned, highly complete dataset (97% matrix, 138 loci, 39,012 bp) and a fixed tree topology as obtained from the 75% matrix. Boxes on the right indicate current distributions color coded to match the regions outlined on the map inset. Reconstructions of likely geographic distributions of ancestors were performed using the best-fitting BAYAREALIKE+j+w model. Fossil taxa were included in the analysis, the gray box indicating their age and the dotted line indicating their hypothesized position in the phylogeny. The vertical gray lines indicate three time slices representing different time periods potentially impacting ancestral distributions. A zoomable figure with all terminal labels can be found in Additional file 1: Fig. S11. **B** Results from Bayesian Stochastic Mapping (BSM) to summarize the number of dispersal events between different regions. The number of inferred events averaged over 100 simulated histories is shown above the arrows and as arrow width

The analysis indicated that Solenostomidae and Syngnathidae split in the late Cretaceous (median 72.06 Ma, 95% highest posterior density interval [HPD] 63.36–80.58) (Fig. 5A, fully labeled tree in Additional file 1: Fig. S11), followed by the Paleocene divergence of Nerophinae and Syngnathinae (median 58.29 Ma, 95% HPD 49.90–67.11). This estimate for the age of Syngnathidae was relatively robust when the oldest known syngnathiform fossil was excluded (median 55.26 Ma, 95% HPD 47.07–63.55) and ca. 5 million years older when all syngnathid fossils were excluded (median 63.67 Ma, 95% HPD 53.90-73.59). In Syngnathinae, the branches leading to the main tribes bifurcated in short time intervals indicating a rapid diversification of the main lineages, while the branching sequence in Nerophinae was less compressed (Fig. 5A).

We estimate the age of the divergence of non-pygmy seahorses and pygmy seahorses to the early Miocene (median 20.80 Ma, 95% HPD 15.97–25.86). Within the non-pygmy seahorse clade, *H. jugumus* split ca. 16.97 Ma (median, 95% HPD 13.24–21.22) from the remaining species. Previous phylogenomic studies were missing pygmy seahorses and *H. jugumus*, and reported conflicting age estimates for this non-pygmy subclade of *Hippocampus *[11, 18]. When excluding the two seahorse fossils, the estimate for the age of this non-pygmy seahorse clade changed only little from 13.66 Ma (median, 95% HPD 11.65–16.39, Fig. 5A) to 15.24 Ma (median, 95% HPD 11.35–19.33, Additional file 1: Fig. S12), indicating that the placement of the seahorse fossils was not significantly driving the age estimate of this clade.

### Historical biogeography

Among the biogeographic models tested for ancestral range reconstruction, the BAYAREALIKE+j+w model produced the best likelihood and highest AIC scores (Additional file 1: Table S6). The deepest nodes of many early syngnathiform and syngnathid lineages were reconstructed to likely have orginated in the former Tethys Sea (Fig. 5A). Inclusion of three Nerophinae fossils from the former Tethys Sea resulted in many early lineages inferred to be of Tethyan origin. Subsequent dispersal events were inferred to the Central Indo-Pacific (ancestors of Microphini and Doryrhamphini), eastern Atlantic (Nerophini), and temperate Australasia (*Leptoichthys* in Nerophini, *Heraldia+Maroubra*).

Ancestors of the earliest Syngnathinae lineages were inferred to be of likely temperate Australian origin (Fig. 5A). It should be noted that no fossils are available for this part of the tree, which could provide historical information for these early lineages. Solegnathini and Stigmatoporini originated and diversified largely in temperate Australia, as did the monotypic and phylogenetically isolated *Stipecampus cristatus*. Leptonotini was inferred to have a Central Indo-Pacific origin with multiple movements to temperate Australasia, of which one lineage diversified into a large, exclusively temperate Australasian clade. Syngnathini also originated in the Central Indo-Pacific and later colonized the Atlantic, where they greatly diversified and crossed back to the Pacific likely through the former Central American Seaway. Haliichthyini colonized temperate Australia a number of times after a Central Indo-Pacific origin. *Halicampus macrorhynchus* and *H. punctatus* and all early lineages of seahorses were inferred to have originated in the Central Pacific. This hypothesis was inferred despite the evidence from the fossil taxa of Haliichthyini and *Hippocampus*, which occupied the Tethys Sea shortly after its separation from the Pacific in the mid-Miocene.

Quantification of dispersal events using Bayesian Stochastic Mapping (BSM) showed a largely asymmetric map of dispersal centered around the Central Indo-Pacific (Fig. 5B). The Central Indo-Pacific was a major exporter of lineages to the adjacent tropical areas in the west (Western Indian Ocean, mean = 26.6 lineages), and to the east (Central Pacific, mean = 19.6 lineages). Temperate Australasia received lineages from the Central Indo-Pacific (mean = 10.5 lineages) but it also moved lineages into the Central Indo-Pacific (mean = 7.5 lineages) at a notably higher proportion than the other regions.

Exchange between the Pacific and the Atlantic was inferred as relatively rare (Fig. 5B). For instance, the exchange between the Western Atlantic and the Eastern Pacific happened prior to the closure of the Central American Seaway, with a higher proportion going west (mean = 5.3 lineages) than east (mean = 3.2 lineages). Syngnathini members share close relatives in the Eastern Pacific (at least 4 exchanges) that were likely separated before the Central American Seaway closed completely. We infer on average seven events of exchange between the Pacific and the Atlantic, of which four occurred in the speciose genus *Syngnathus* and in a subgroup of *Hippocampus*. These two groups originated around a similar time but in different ocean basins: *Syngnathus* is reconstructed to have originated ca. 13.06 Ma in the Western Atlantic and has since then exported at least two lineages to the Pacific before the final closure of the Central American Seaway. The main seahorse clade (i.e., the sister group to *H. jugumus*) is reconstructed to have arisen ca. 13.66 Ma in the Central Indo-Pacific and has since exported two lineages to the Atlantic, which further diversified there.

Dispersal events crossing the Atlantic were more common from the Western Atlantic to the Eastern Atlantic (mean = 5.8 lineages) than vice versa (mean = 3.2 lineages, Fig. 5B). Much of Syngnathini was reconstructed to have originated in the Western Atlantic and eastward trans-Atlantic dispersal was inferred twice (one leading to *Enneacampus*, one within *Syngnathus*) with subsequent speciation in the Eastern Atlantic. An example of trans-Atlantic dispersal from east to west was inferred to have occurred in the clade of *Microphis aculeatus* and *M. lineatus* and two such events happened in seahorses.

### Diversification rate analysis

Diversification rates of Syngnathidae and Solenostomidae were heterogeneous between geographic regions (Additional file 1: Fig. S13). Between ocean basins, the mean age of species was younger in the Atlantic than the Pacific (Atlantic mean = 4.99 Ma, Pacific mean = 7.84 Ma, Welch two sample* t*-test, *P* value = 6.71e−5), indicating more recent colonization and speciation within the Atlantic. Estimates of tip rates of species-specific speciation were higher in the Atlantic than in the Pacific Ocean, both for the DR statistic (Atlantic mean = 0.15 species/Ma, Pacific mean = 0.10 species/Ma, Welch two sample *t*-test, *P* value = 3.46e−4) and tip diversification rates from ClaDS (Atlantic mean = 0.14, Pacific mean = 0.10, *P* value = 6.24e−6), indicating more actively diversifying lineages in the Atlantic.

**Fig. 6:**
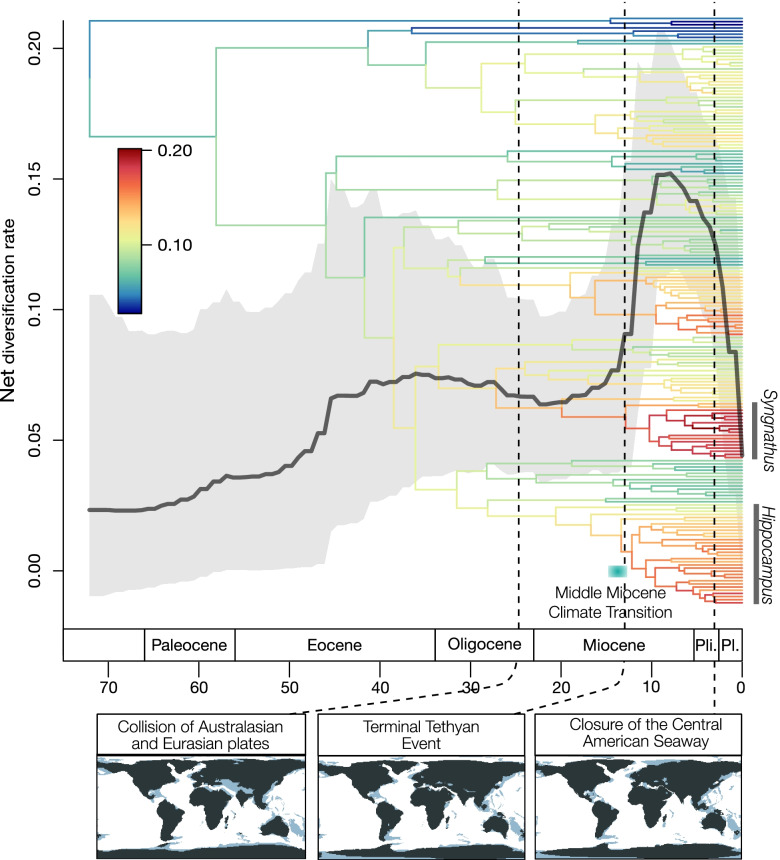
Diversification of Syngnathidae and Solenostomidae through time. The colors on the phylogenetic tree show branch-specific estimates of net diversification rates (i.e., speciation rate minus extinction rate) from ClaDS. The curve is showing tree-wide net diversification rate estimated using an episodic birth–death model in RevBayes. The mean estimate is the gray line, and the light gray shading represents 95% HPD intervals. The vertical dashed lines indicate major geographic events, which are illustrated in the maps below with reconstructions of shallow seafloor areas in light blue (plotted with data from [64])

Diversification rates also varied through time. Under an episodic birth–death model accounting for incomplete sampling, we found that net diversification rate (i.e., speciation rate minus extinction rate) varied through time between 0.02 and 0.15 species/Ma (Fig. 6). After a relatively slow increase of the diversification rate from the origin of the clade to the middle Eocene, diversification rates increased ca. 45 Ma, leveled off and decreased slightly in the Oligocene. In the Miocene, a fast and steep increase in diversification rate started ca. 19 Ma, roughly doubled to peak ca. 9 Ma ago, and then decreased to the present (Fig. 6).

This variation in diversification rates appeared to be largely driven by speciation rate increases within subclades of *Syngnathus, Hippocampus* and, to a lesser degree, the Australian clade of Leptonotini (Fig. 6). These three groups contained species that were among the lineages with the fastest rates of DR and tip diversification rates (Additional file 1: Fig. S14). On the branch leading to extant species of *Syngnathus* diversification rates increased to an intermediate value in the two species occurring in the East Pacific and then increased in their globally distributed sister group. Within *Hippocampus*, diversification rates were lower in pygmy seahorses, in the branch leading to *H. jugumus*, and then increased in the globally distributed seahorse subclade.

## Discussion

In this study, we have produced a substantial phylogenomic dataset for Syngnathidae (1314 UCEs, 303 individuals, 183 species), representing 93% of genera and 62% of the extant species. The extended sampling revealed the major organization of syngnathids in nine tribes, showed several taxonomic problems, and  proposed a new sister group of seahorses. We provide a comprehensive review of the under-explored fossil record, including nine with newly proposed phylogenetic placements, which were used to calibrate the phylogenetic tree. We used this phylogenetic hypothesis to infer ancestral biogeographic regions and found unexpectedly frequent exchanges between the temperate coast of Australasia and the tropical Indo-Pacific. Lastly, we inferred a pronounced increase in diversification rates in the late Miocene that appears to be largely driven by rapid speciation of seahorses and *Syngnathus* pipefishes.

### Taxonomic implications

We found a number of instances of taxonomic uncertainty, including nine non-monophyletic genera, five species that were previously synonyms of other species, and seven potentially undescribed species (Fig. 2, Additional file 1: Taxonomy). Genetic assessment of multiple individuals expanded the known distributional ranges for nine species. This basic information on species status and ranges are important given that syngnathids are globally traded [65] and are of conservation concern [66, 67].

The extensive non-monophyly reveals that several characters that have been used to delimit genera have evolved multiple times. Generic characteristics are usually associated with shapes of the snouts, snout ridges, and organization of the bony external skeleton [43]. For example, our results robustly confirm that seahorse-like features, such as the prehensile tail, were independently acquired at least three times in the Indo-Pacific pygmy pipehorse genera *Idiotropiscis*, *Acentronura*, and *Cylix* (Haliichthyini), the Atlantic pygmy pipehorse *Amphelikturus* (Syngnathini), and in seahorses (*Hippocampus*) [44]. These independently evolved characters refute earlier studies of a close sister relationship between seahorses and pygmy pipehorses [52, 54]. Gaining a better understanding of how skeletal elements evolved is of importance because syngnathids are models for suction feeding [68] and for bioinspired armor [5].

Our analysis identified a new sister group to seahorses, a clade of the Indo-Pacific pipefish species *Halicampus punctatus* and *H. macrorhynchus*. This finding is surprising given that no obvious morphological similarities between these two groups have been identified in the literature. Although *H. macrorhynchus* was previously recovered in the vicinity of seahorses as a close sister to members of the Haliichthyini tribe, the support was low and inconsistent [44]. This new sister relationship is of significance because it suggests that the highly modified morphologies of seahorses were derived from a pipefish-like ancestor, rather than from the superficially intermediate form of a pipehorse ancestor. As we are learning about the genetic mechanisms underlying syngnathid phenotypes [6, 7], information about the closest living relatives of seahorses, seadragons, and various pipefishes will be important.

### Fossil Syngnathiformes

The denser species sampling provided the opportunity to review and formulate new hypotheses on the phylogenetic affinities for several syngnathiform fossils (Additional file 1: Fossil Justifications), which gives a more complete picture of extinct diversity, ancient biogeographic ranges, and the ages of groups. We also identified a number of equivalent fossils which calibrate the same node as other fossils, which may in the future benefit dating approaches that draw on the distribution of fossil records [69], not just the oldest known fossils as used here.

Particularly within Syngnathidae, a number of fossils are well preserved and described [47, 48, 70] but have not been used extensively in evolutionary analyses. Because the extant species relationships clearly indicate that morphological traits evolved often convergently in syngnathids, this obviously could extend to the traits of the fossils. We therefore excluded sets of fossils to confirm that the age estimates were robust. Another approach would be to use total-evidence dating [71] including a morphological matrix for both extant and extinct taxa with sufficient characters to tolerate potential convergent traits. However, such an approach is currently limited by the lack of comprehensive morphological data for Syngnathidae and Syngnathiformes. We note the opportunity for many more syngnathid fossils to be incorporated in the future, particularly within tail brooding Syngnathinae, when the evolution of their skeletal traits is better understood.

### Improved time calibration

Our estimates for the evolution of syngnathiform lineages were largely congruent with the most recent study [11], but our extended fossil catalog allowed testing the effect of including different sets of calibration points. We were specifically interested in the age of the most recent common ancestor of Syngnathidae, which was here estimated to the Paleocene, ca. 58.29 Ma (Fig. 5A), somewhat younger than the most recent study that used only one seahorse fossil and a geological calibration 11. We found that excluding all seven syngnathid fossils brought the median estimate to 63.67 Ma, closer to the most recent estimate. The additional information on ages of certain nodes therefore resulted in a somewhat younger age estimate of the origin of syngnathids.

We also tested the effect of omitting the seahorse calibrations, which have been differently interpreted by previous studies and ours. While one study placed the mid-Miocene fossil †*H. slovenicus* in close relation to the extant *H. trimaculatus*, which pushed the age of the main group of non-pygmy seahorses to ca. 24 Ma in the late Oligocene [49], another study placed the same fossil on the crown of the main group of non-pygmy seahorses and estimated an age of 14 Ma [11]. Here, we again have a different interpretation of this fossil as a crown member of all *Hippocampus* including pygmy seahorses (Additional file 1: Fossil Justifications). Rather than arguing for one placement of the fossil over the other, we found that omitting the fossils only marginally changed the inferred ages of the main seahorse lineages (Additional file 1: Fig. S12): The node in conflict was inferred to be 15.24 Ma old without seahorse calibrations, and 13.66 Ma including the calibrations. Overall these results align more closely with [11] and refute a late Oligocene origin of the main group of non-pygmy seahorses. As opposed to the other two studies, we also include *H. jugumus* and pygmy seahorses, which are deeper lineages of *Hippocampus* than the main group of non-pygmy seahorses. The age of this entire *Hippocampus* clade is estimated to ca. 20.80 Ma. The improved fossil representation and taxon sampling therefore provides a more complete picture of the origin and diversification of seahorses.

### Diversity and endemism

The Central Indo-Pacific hosts, by far, the highest number of living syngnathid and solenostomid species in addition to significant levels of phylogenetic diversity (Fig. 4), highlighting once again the importance of this region for fish diversity [72]. We also inferred it to be the principal area where syngnathid species originate and emigrate from (Fig. 5b). This role of the tropical Central Indo-Pacific as a generator and exporter of lineages has been described in various reef fishes [73].

Our results also show that the southern coast of Australia and New Zealand has high phylogenetic diversity, and particularly stands out as a hotspot of phylogenetic endemism (Fig. 4). These temperate waters thus harbor a significant proportion of evolutionary unique lineages that occur only in these regions. Temperate Australasia is known for its high endemism of marine groups [55]. The endemicity is often explained by the long-term temporal and spatial isolation from other coastal areas allowing for increased persistence of lineages; nearby archipelagos and islands are separated by deep trenches or basins, restricting the migration of low dispersal species such as syngnathids. These endemics also highlight the significance of temperate regions, not just tropical regions, as important reservoirs of evolutionary history.

A number of lineages were also exported from the Australasian region to the Central Indo-Pacific (Fig. 5b). While the absolute number of lineage exchanges across the tropical parts of the Pacific was found to be higher, the exchanges between the temperate Australasian and the tropical Indo-Pacific regions stand out because they are more symmetrical. This means that the temperate Australasian region not only received lineages from the Central Indo-Pacific but also returned a number of lineages. This exchange between tropical and temperate regions in syngnathids stands in contrast with patterns seen in many reef fishes, where dispersal rates for tropical lineages expanding into temperate areas are higher than vice versa [74, 75]. While the majority of syngnathid lineages have arisen from tropical ancestors, following the ‘out-of-the-tropics’ model [76], the temperate Australasian region has contributed to the syngnathid species diversity in the Central Indo-Pacific.

### Faunal origins and colonization pathways

The inferred origin of early ancestors of Syngnathiformes and Syngnathidae in the former Tethys Sea agrees with a number of other marine taxa [77]. The inclusion of fossils is crucial for this inference because an occurrence in the now vanished Tethys Sea cannot be deduced from distributions of extant taxa only [78]. For example, a previous study supporting a Central Indo-Pacific origin of Syngnathidae and the early lineages was likely due to only one syngnathid fossil used in that study [11]. In contrast, we find a Tethyan origin of Syngnathidae and of the first lineages of Nerophinae, likely due to the inclusion of three Tethyan fossils belonging to Nerophinae. If Tethyan fossils were also available for early tail brooders, the inferred diversification of early Syngnathinae could also change.

Dispersal out of the Tethys Sea to the Central Indo-Pacific was inferred for syngnathiform lineages (Fig. 5a) and in one of the ancestors of Microphini and Doryrhamphini, mirroring the movement of a hotspot of biodiversity from the Tethys Sea to the Central Indo-Pacific [79]. However, dispersal from the Tethys Sea to the (now temperate) Australasian region was also inferred. These reconstructed early dispersal events between the Eurasian coast (Tethys Sea or Central Indo-Pacific) and the Australian coast are somewhat puzzling because the Australian continent used to be located 3000 km further south than today and was widely separated from the Central Indo-Pacific [80]. Only after Australia started moving northwards and collided with the Eurasian plate ca. 25 Ma [81] was lineage exchange without requiring long-distance dispersal possible through the modern Central Indo-Pacific route. Despite parameterizing the biogeographic model against frequent exchanges to Australia before 25 Ma (Additional file 1: Table S5), temperate Australian taxa such as *Stipecampus*, Solegnathini, and Stigmatoporini are lineages with reconstructed Australian origin before the continent had moved north (Fig. 4a).

Similar old phylogenetic connections between Australian and Tethyan faunas have been proposed for skates [82] and for labrid fishes [64]. Plate tectonic reconstructions suggest a now submerged route may have existed in the form of an island network in the Southern Indian Ocean formed by volcanic hotspot systems [83]. These islands appear to have served as stepping stones for dispersal of terrestrial and marine taxa between Madagascar and Eurasia [84, 85] and possibly between the Western Indian Ocean and the Australian continent [64, 86–88]. The syngnathid examples add to the evidence that an Australian endemic marine fauna appears to have existed before the Central Indo-Pacific hotspot formed.

### Increased diversification rates in the Miocene

The diversification rates inferred from the phylogeny of Syngnathidae and Solenostomidae showed a marked increase and subsequent decrease in the Miocene (Fig. 6). This increase was largely driven by three clades that diversified in different regions of the world: seahorses in the Central Indo-Pacific, *Syngnathus* in the Western Atlantic, and the subclade of Leptonotini in Australasia.

This simultaneous expansion in different ocean basins raises the possibility of environmental factors that influenced higher speciation rates at a global scale. However, the inferred changes happened during a time of significant global changes in climate and shallow habitat availability [89], which could have all impacted syngnathid speciation and extinction. Increased diversification rates observed in shallow-water gastropods and reef-building corals in the Indo-Pacific are attributed to increased habitat availability after the collision of the Australasian and Eurasian plates [64, 90]. However, the increased rate of diversification observed here started well after the collision and these Indo-Pacific changes would not explain the diversification of *Syngnathus* in the Western Atlantic.

The inferred diversification rates of syngnathids were fastest around the time of the Middle Miocene Climate Transition (14.5–12.5 Ma) and the Terminal Tethyan Event (13.8 Ma) (Fig. 6). During the Middle Miocene Climate Transition, sea surface temperatures cooled differentially across the Atlantic, resulting in a 4-million-year-long absence of the typical latitudinal sea surface temperature gradient [91]. It is possible that this absence of a temperature gradient facilitated range expansions of Atlantic syngnathids, which subsequently became isolated and speciated. Therefore, it might explain the rapid diversification observed among the members of *Syngnathus*, which were inferred to be of Western Atlantic origin, but not among *Hippocampus* species, which are of Central Indo-Pacific origin. The decrease in diversification rate since the late Miocene into the Plio-Pleistocene coincides with general cooling over the past 12 Ma and the onset of strong, frequent sea level fluctuations that started ca. 8.9 Ma [89].

An alternative explanation for the heightened speciation rates could be a biological propensity to disperse more easily. A significant feature of *Hippocampus* and *Syngnathus* clades is that they appear to have dispersed a number of times between the Atlantic and the Pacific (Fig. 5a). These long-distance dispersal events to a new location and subsequent speciation likely contributed to the heightened diversification observed in these clades. At least seahorses are known to be able to raft on detached floating kelp away from shore [92], which could facilitate long distance dispersal. We found evidence that west to east dispersal in the Atlantic was more common than the other direction (6 compared to 3 dispersals), consistent with the direction of the Gulf Stream. However, this explanation does not account for the heightened diversification rate observed in the subclade of Leptonotini, which has no inferred dispersal events.

## Conclusions

This well-sampled, well-supported phylogenomic hypothesis for Syngnathidae, together with data for other Syngnathiformes and their fossil taxa, provides a new framework in which to study the evolution of this group, especially the evolution of their reproductive and morphological traits. We found that the group diversified in the Cenozoic, reaching shoreline habitats around the world with an unusual propensity to return to tropical regions from temperate regions. Increased diversification rates in the Miocene may be linked to global temperature changes and to exchanges between Atlantic and Pacific faunas. We conclude that densely sampling taxa for genomic regions, including fossil taxa and biogeographic information, can provide a unified view of the evolutionary processes associated with the diversification and the global colonization routes of marine organisms.

## Methods

### Sampling design and UCE sequencing

Our goal was to densely sample the diversity of Syngnathidae; we achieved this through acquiring a total of 183 species and 303 specimens (Additional file 2). We included 57 species and 59 specimens of Syngnathidae from a previous study [9] from raw sequence reads available from NCBI [93]. Compared to the currently best sampled phylogeny with few loci (91 species, 48 genera [44]), we added 92 species, five genera (*Campichthys*, *Doryichthys, Ichthyocampus*, *Leptonotus*, and *Minyichthys*), in addition to the recently described genus and species *Cylix tupareomanaia* [63]. Of these, *Doryichthys* has been included in [9]. Our sampling covers 54 of 58 genera and 183 of 296 of recognized species. Four pipefish genera were not sampled, including *Bulbonaricus* (contains three species) and three monotypic genera (*Kimbaleus, Kyonemichtys, Notiocampus*). We included multiple specimens for 39% (71/183) of the sampled species to assess intraspecific relationships, focusing on species with wide geographic ranges or problematic taxa identified by previous work.

In order to facilitate time calibration by taking advantage of the range of fossils in Syngnathiformes, we extended our sampling to also include UCEs from an earlier study (113 species including one Scombriformes outgroup, [9]). The combined total from both studies amounts to 363 individuals representing 238 species.

We extracted DNA from frozen or dried tissues using the Qiagen DNeasy Blood and Tissue Kit (Qiagen), quantified using a Qubit fluorometer (Life Technologies, Inc.). For 15 low DNA quantity samples, we used illustra Ready-To-Go GenomiPhi v3 DNA amplification kits (GE Healthcare Life Sciences), which representatively amplifies low-quantity DNA. DNA was randomly sheared by sonication with a Bioruptor Standard (Diagenode Inc.) into fragments of an average size of 400−700 bp. Short-read sequencing libraries were prepared using commercial kits (Kapa Biosystems, Inc.) and dual indexes [94] with an input of 60–1200 ng DNA (average 900 ng). We used a SPRI beads substitute for clean-up steps [95]. Before sequencing, we enriched pools of sequencing libraries for 1314 UCE loci using commercially synthesized baits ([58], Mycroarray MYbaits Kit, Acanthomorphs 1Kv1 https://ndownloader.figshare.com/files/11188235). After enrichment, we used 16–18 PCR cycles to recover enriched loci. Sequencing of 233 samples was done using 100 base pair paired-end (bp PE) sequencing on a shared lane of HiSeq4000 (Illumina, Inc.) and 15 samples were sequenced on MiSeq (Illumina, Inc.) runs with 300 bp PE v3 chemistry and 250 bp PE v2 chemistry.

https://dataview.ncbi.nlm.nih.gov/object/PRJNA734786?reviewer=nvgcallj7r51j285g69ni21eu7

### Data processing

Computation was performed on the National Life Science Supercomputing Center - Computerome 2.0 (www.computerome.dk) and the computational resources of the Center for Comparative Genomics at the California Academy of Sciences. Newly sequenced samples and published samples were processed in the same manner. Paired-end raw sequences were processed with fastp [96], which trims adapters, low-quality bases, ambiguous base calls, and removes low-quality reads. Subsequent processing was done with functions of phyluce [97] and standard protocols were employed unless specifically mentioned (https://phyluce.readthedocs.io/en/latest/tutorials/tutorial-1.html). We tested two assemblers, abyss v.1.5.2 [98] and spades v.3.14 [99]. In most cases, the assemblers produced similar assemblies; however, for some samples, strong variability was observed in the number of contigs produced. We therefore assembled sequences of each sample using both programs and selected the assemblies with the highest number of UCE loci (Additional file 2). Loci were aligned with MAFFT v.7.475 [100] and trimmed with Gblocks v.0.91b [101]. We also used BLASTN v. 2.8.1+ to compare publicly available mitochondrial barcodes [102] against contigs present in some of the best assemblies to confirm species identifications.

### Phylogenetic analyses

We used concatenation- and coalescent-based species tree analyses to estimate phylogenetic relationships. We investigated two main datasets: (1) a dataset containing multiple individuals for certain species (361 individuals, 238 species of Syngnathiformes); and (2) a dataset containing only one individual per species (238 individuals and species), for which we selected the individual that had the most sequenced UCEs. For both datasets, we generated a concatenated matrix from alignments that were present in at least 75% and 90% of the individuals. Trees were estimated using maximum likelihood with IQ-TREE2 v.2.1.2 [103] using the most appropriate nucleotide substitution model as selected by ModelFinder [104] on the entire alignment and 1000 ultrafast bootstrap replicates [105]. For coalescent-based analyses, we estimated individual gene trees using all 361 samples and 1312 alignments using the same commands as for concatenation and summarized the gene trees using ASTRAL-III v.5.15.4 [106].

### Fossil calibrations

We carefully reviewed available fossil information for Syngnathiformes and provide justifications for their hypothesized phylogenetic affiliation based on shared apomorphies for 14 syngnathiform fossils, half of them in Syngnathidae (Additional file 1: Fossil Justifications; Fig. 4). Nine fossils were used for the first time or with newly proposed relationships. In Syngnathidae, we identified three fossils in Nerophinae, two in Haliichthyini, and two in *Hippocampus*.

We used fossils as node-based calibrations, where the oldest fossil of a clade was used as a constraint to specific nodes. Fossil calibrations can only provide a lower bound on the age of a clade of interest, not an upper bound for the clade age. In order to avoid arbitrary upper bounds, we used an approach that defines the calibration density of the prior based on an outgroup sequence [107], which has been developed for fishes [58, 108] (Additional file 1: Calibration Densities).

We investigated the impact of using different sets of fossils on the divergence analysis. Specifically, (1) we removed the fossil constraint on the stem Syngnathoidei (*†Gasterorhamphosus zuppichini*) to test for the impacts of the recently proposed older age (ca. 83.6 Ma) of the fossil [11] than previously used in other studies [58, 109, 110]; (2) we removed all Syngnathidae and Solenostomidae calibrations (7 constraints) to investigate the impact of these less established, newly defined constraints; and (3) we removed calibrations within *Hippocampus* (2 constraints, †*H. slovenicus,* †*H. sarmaticus*) to investigate the discrepancy that two recent studies obtained for the age of the main group of the non-pygmy seahorses (24 Ma [49] or 14 Ma [11]). We therefore assess the impact that the removal of these controversial fossil constraints has on the inferred age of the main group of non-pygmy seahorses.

### Divergence time analysis

To reduce the computational burden of divergence time estimation with BEAST2 v.2.6.3 [111], we used highly complete alignments that were present in 97% of the species (138 loci, 39,012 nucleotides). Because partitioning for each locus is susceptible to overparameterization on the relatively short loci, we used ModelFinder [104] to merge similar partitions with a procedure akin to PartitionFinder [112]. The resulting 12 partitions were then used by BEAST2’s bModelTest package to average over nucleotide substitution models for each partition [113]. We used the tree obtained from the larger 75% matrix as a topological constraint throughout the analysis. The BEAST2 analyses were performed using a birth–death model and with a fast uncorrelated lognormal relaxed molecular clock [114]. Analyses were run for 50 million generations with samples drawn every 5000 generations and three independent analyses were run from different starting seeds. Analyses were checked for convergence in Tracer v.1.7.1 [115] and the effective sample size (ESS) values for each parameter were all >200. LogCombiner and TreeAnnotator of the BEAST2 package were used to summarize the three runs after a burnin of 30% each and to generate a maximum clade credibility (MCC) tree. Plotting of comparisons of different dated trees was done with the compare.phylo function of phyloch (https://rdrr.io/github/fmichonneau/phyloch/), and posterior calibration densities were plotted with the MCMC.tree.plot function of MCMCtreeR (https://github.com/PuttickMacroevolution/MCMCtreeR) using 100 post-burnin posterior trees from BEAST2.

### Diversity and endemism

We generated an updated list of extant species of Syngnathidae and Solenostomidae, which was based on seminal works on the taxonomy [43, 51, 116], with updates of recently described and synonymized species (Additional file 1: Table S1). In order to quantify the spatial distribution of diversity, independent of predefined geographic regions, we calculated metrics that quantify the shared evolutionary history of species diversity on a geographic grid. We used the R package PDcalc (https://github.com/davidnipperess/PDcalc) to calculate phylogenetic diversity, which summarizes the length of branches linking taxa in a particular geographic region [117], and phylogenetic endemism, which identifies grid cells where substantial components of phylogenetic diversity are restricted [118]. We used spatial polygons outlining the distribution of 263 species of Syngnathidae and Solenostomidae compiled by the IUCN SSC Seahorse, Pipefish & Seadragon Specialist Group [119]. We used the subset of species that matched terminals in our phylogeny (161 species) and transformed the range polygons to a presence/absence matrix at a 2° resolution with the R package letsR [120]. Grid cells were required to have at least two occupying species to be included in the calculation.

### Biogeographic reconstruction

In order to reconstruct historical biogeography using the timetree of Syngnathiformes, we performed ancestral area estimates in the R package BioGeoBEARS v.1.1.2 [121]. Geographical ranges for each species were obtained from the literature [43, 51, 116] and from our geographic sampling (Additional file 1: Taxonomy). All extant species were coded as absent or present in seven major biogeographic realms: Western Atlantic; Eastern Atlantic; Western Indian Ocean; Central Indo-Pacific; Central Pacific; Eastern Pacific; and Temperate Australasia. This delineation follows [122], but with temperate Australia and New Zealand separated because these areas host significant syngnathid diversity [43, 44]. We also compared this biogeographic delimitation to an algorithmic delimitation that is based on range data for taxa of interest [123] (https://www.mapequation.org/bioregions/, settings: maximum and minimum cell size 1 degree, cell capacity 5–100, cost 2.5, 5 trials). Using the range polygons as above resulted in a similar delimitation with the exception that the Western Indian Ocean, Central Indo-Pacific, and the Central Pacific were grouped into a single region (Additional file 1: Fig. S15).

We added the former Tethys Sea as an eighth, now extinct region to account for the known occurrence of fossil Syngnathiformes. The R packages phytools [124] and ape [125] were used to add fossils to the timetree in their hypothesized positions (14 fossil calibrations plus 1 fossil, which was redundant for node-dating; Additional file 1: Fossil Justifications).

Connectivity of these biogeographic areas was modeled with three dispersal probability categories: 1.0 for well-connected adjacent areas; 0.1 for areas requiring trans-oceanic dispersal; and 0.001 for areas that are separated by land or by another biogeographic region (Additional file 1: Table S5). Allowed areas were set to adjacent areas with a maximum of five at a given node, which is the number of areas a few widespread Syngnathiformes occupy. Area connectivity and dispersal probability were modeled in four time slices to account for geological events that influenced shallow marine organisms: (1) root to 25.0 Ma, during which connectivity between Australasia and other areas was modeled as trans-oceanic dispersal because Australia was located ca. 3000 km further south than today [80]; (2) 25.0 to 13.8 Ma, during which Australasia and Central Indo-Pacific were modeled as well-connected after the Australian Plate collided with the Eurasian Plate [81]; (3) 13.8 to 3.5 Ma, during which the Atlantic and Indo-Pacific areas were modeled as separated after the Terminal Tethyan Event [126], yet a low dispersal multiplier value (parameter +w) was kept between the Western Indian Ocean and the East Atlantic to allow the possibility of dispersal around southern Africa [127], and (4) 3.5 to 0 Ma, during which the Western Atlantic and East Pacific regions were as separated due to the closure of the Central American Seaway [128].

We compared ancestral area estimates using the DEC, DIVALIKE, and BAYAREALIKE models and the same set of models including the +j parameter [129] and the +w parameter [130]. Accounting for potential founder speciation with the +j parameter may be relevant for syngnathids because at least seahorses are known to occasionally disperse through rafting attached to floating vegetation [92]. The +w parameter is the exponent to the dispersal probabilities that reduces the subjectivity in the dispersal multipliers. The resulting models were compared using AIC scores.

To quantify the number of dispersal events between different areas across the tree, we used the best-fitting model to perform Bayesian Stochastic Mapping (BSM) using 100 simulated histories of possible changes along branches while taking uncertainty into account [130]. We averaged the number of lineages that were exported out of each region or imported into each region.

### Diversification rate analysis

In order to investigate diversification patterns for Syngnathidae and Solenostomidae, we estimated tip-specific speciation or diversification rates with three metrics for geographic regions and ocean basins. First, we calculated the age of the terminal branches (i.e., the age of species since their split from their most recent common ancestor) using the R package picante [131]. Second, we calculated speciation rates using the non-parametric approach of the DR statistic [132] in picante. Third, we estimated tip diversification rates using the model-based approach of ClaDS [133]. ClaDS provides tip-specific as well as diversification rates across the branches of the tree, estimated using the implementation [134] for Julia v.1.5.1. Rather than assuming that speciation rates are taken from a small set of rate regimes, ClaDS assigns unique speciation rates to each lineage, which are then inherited by the daughter lineages. This allows ClaDS to detect frequent speciation rate shifts with small effects, making it particularly relevant for estimating species-specific rates [133]. In order to account for incomplete sampling, we used sampling probabilities for each tip (Additional file 1: Table S1). Missing species were assigned to their genus. For non-monophyletic genera, we assigned missing species based on taxonomic characters, while members of the four missing genera were distributed across the entire Syngnathidae because their phylogenetic placement remains to be established.

To investigate diversification rates through time across the phylogeny, we used an episodic birth–death model, in which speciation and extinction rates are allowed to vary between time intervals [135]. We used RevBayes v.1.1.1 [136] and a Horseshoe Markov random field (HSMRF) prior distribution on log transformed rates [137] with 100 time intervals. This prior assumes that rates are autocorrelated, meaning that the rates in the current time interval are informed by the previous one. We accounted for incomplete sampling using clade-specific sampling probabilities [138] as described above (Additional file 1: Table S1).

## Supplementary Information

**Additional file 1: **Calibration Densities, Fossil Justifications, Systematics, Taxonomy – Detailed information on calibration densities, justification of fossil node calibrations, and details on systematic and taxonomic findings. **Figures S1-S7.** Phylogenetic hypotheses obtained from different concatenation and coalescent-based analyses. **Figures S8-S12.** Calibration densities and impact of different sets of fossil calibrations on age estimates. **Figures S13-S14.** Estimates for diversification metrics for biogeographic regions and across the phylogeny. **FigS15.** Results from algorithmic delimitation of biogeographic regions. **Table S1.** Number of described species for each genus of Syngnathidae and Solenostomidae and proportion sampled in this study. **Table S2.** Comparison of fossils and extant genera of Nerophinae. **Table S3.** Overview of sampled specimens and species in the present study and three recent studies. **Table S4.** Statistics of matrices analyses for phylogenetic reconstruction. **Tables S5-S6.** Matrices of dispersal multipliers for different time periods and parameters and estimates from biogeographic reconstruction.

**Additional file 2.** Table of sampling data for all included specimens.

## Data Availability

The datasets generated and analyzed in this study are included in this published article and its supplementary information files and in the FigShare repository, 10.6084/m9.figshare.19232793.v1 [139]. The repository contains the assemblies, alignments, log files, and tree files from phylogenetic analysis and dating analyses, calculations for phylogenetic diversity and endemism, and results from BLAST searches against publicly available COI barcodes for species confirmation. Raw sequence reads are available from NCBI (BioProject PRJNA734786, https://identifiers.org/bioproject:PRJNA734786) [140]. New tribe names have been registered under ZooBank (urn:lsid:zoobank.org:pub:3D1F608C-B8B0-4C8E-B2DD-E0F88D73E09B).
