# Supplementary material for: Phylogenomic analysis of Syngnathidae reveals novel relationships, origins of endemic diversity and variable diversification rates
Source: BMC Biol. 2022 Mar 27;20:75. doi: 10.1186/s12915-022-01271-w (PMC8962102; doi:10.1186/s12915-022-01271-w)
Supplement: Supplementary file 1 — Additional file 1: Calibration Densities, Fossil Justifications, Systematics, Taxonomy – Detailed information on calibration densities, justification of fossil node calibrations, and details on systematic and taxonomic findings. Figures S1-S7. Phylogenetic hypotheses obtained from different concatenation and coalescent-based analyses. Figures S8-S12. Calibration densities and impact of different sets of fossil calibrations on age estimates. Figures S13-S14. Estimates for diversification metrics for biogeographic regions and across the phylogeny. FigS15. Results from algorithmic delimitation of biogeographic regions. Table S1. Number of described species for each genus of Syngnathidae and Solenostomidae and proportion sampled in this study. Table S2. Comparison of fossils and extant genera of Nerophinae. Table S3. Overview of sampled specimens and species in the present study and three recent studies. Table S4. Statistics of matrices analyses for phylogenetic reconstruction. Tables S5-S6. Matrices of dispersal multipliers for different time periods and parameters and estimates from biogeographic reconstruction. [file 12915_2022_1271_MOESM1_ESM.pdf]

Additional file 1

Accompanying Stiller, Short, Hamilton, Saarman, Longo, Wainwright, Rouse & Simison. Phylogenomic analysis of Syngnathidae reveals novel relationships, origins of endemic diversity and variable diversification rates. *BMC Biology* 2022.

Table of Contents

Calibration Densities 2

Fossil Justifications 3

Systematics 14

Tribes in Nerophinae 14

Tribes in Syngnathinae 15

Taxonomy 18

(1) Potential new species 18

(2) Synonymized species that are distinct 19

(3) Non-monophyletic genera 20

(4) Extensions of known ranges or confirmed wide distributions 23

Additional Figures 25

Additional Tables 40

Calibration Densities

The Hedman approach [107] generates a distribution of probable time of origin for a node based on ages of a sequence of outgroups, given (i) the minimum age of the calibrated node, (ii) a sequence of stratigraphically consistent of older fossil outgroup to that node, and (iii) a hard maximum bound on the ages. More distant outgroups have to appear earlier than more proximate outgroups to the focal node, i.e. the fossil record is stratigraphically consistent with the tree [107].

The outgroup sequence used here is based on [108] with modifications adopted from [58]. The outgroup sequence is in order of the oldest minimum age: 247.1 Ma, Holostei, †*Wasonulus eugnathoides*; 236.0 Ma, †*Prohalecites porro*i; 221.0 Ma, †Pholidophoridae, †*Knerichthys bronni*; 193.81 Ma, †*Dorsetichthys bechei*; 181.7 Ma, †*Leptolepis coryphaenoides*; 166.1 Ma, †Ichthyodectiformes, †*Occithrissops willsoni*; 151.2 Ma, Elopomorpha, †*Anaethalion zapporum*; 150.94 Ma, Otocephala, †*Tischlingerichthys viholi*; 150.94 Ma, non-eurypterygian Euteleostei †*Leptolepides haerteisi*; 125.0 Ma, Aulopiformes, †*Atolvorator longipectoralis*; 98.0 Ma, Lampridiformes, †*Aipichthys minor*; 98.0 Ma, Holocentroidei, †*Stichocentrus liratus*.

We extended this outgroup sequence with stratigraphically consistent calibrations included in our own set of fossil-based minimum ages for Syngnathiformes. We also include one additional fossil, for which the phylogenetic position could be specified as a crown Syngnathidae but which was not used as calibration itself (see Additional node A). The outgroup sequence for each calibration point is given under “Outgroup age sequence”. The outgroup sequence requires a maximum hard bound, which was set in the Carboniferous (322.8 Ma, stem neopterygian †*Discoserra*, [108]), far exceeding any of the divergences investigated here.

In order to estimate the calibration densities for each of the 14 calibration nodes, we applied the outgroup sequence with an R script from [108]. The obtained mean and 95% confidence interval was applied on the prior distribution of the node age in BEAST2 (specific settings given under “Settings in BEAST” for each node).

Fossil Justifications

Additional Node A: Crown Syngnathidae

Because a central goal of the fossil calibrations was to infer the ages of nodes within Syngnathidae, we did not place an age constraint on the MRCA of Syngnathidae. Instead, we use the information of this crown Syngnathidae fossil as part of the outgroup sequence to define the prior distribution of node ages for syngnathid calibrations (Nodes 8-14).

Fossil taxon: *†Prosolenostomus lessinii* Blot 1980

Material: description of holotype, IG 37597 Vérone.

Phylogenetic placement: MRCA of Syngnathidae

Placement justification: The original description placed the fossil as a stem Solenostomidae [12]. The presence of a number of Syngnathidae features, which are missing in Solenostomidae, is evidence for a placement as a crown Syngnathidae: presence of dermal bony rings (stellate plates in Solenostomidae); single dorsal fin (double in Solenostomidae); pelvic fins absent (present in Solenostomidae) [13, 46, 70]. Because of the absence of apomorphies for Syngnathidae relative to the fossil, it cannot be placed on the stem of Syngnathidae (which would also make it synonymous with Node 7). The presence and location of the brood pouch is unknown, which makes it hard to know if the fossil could be placed on the stem of Nerophinae or Syngnathinae or within one of these groups. We therefore exclude the fossil as a node calibration point but include its information content with respect to middle Eocene occurrences of Syngnathidae in the outgroup sequence for syngnathid calibrations.

Stratigraphy: middle Eocene, Ypresian, Pesciara or Monte Bolca, Italy

Minimum age: 48.5 Ma

Age justification: The deposits from Monte Bolca's two principal Lagerstätten correspond to large parts of the Shallow Benthic Zone (SBZ) 11 of the late Ypresian Stage and have been dated to 50.7-48.5 Ma [14].

Discussion: Equivalent fossils from the same deposit may be "*Syngnathus*" *heckeli* de Zigno 1874a and "*Syngnathus*" *bolcensis* de Zigno 1874b. Their attribution to the extant genus *Syngnathus* should be verified, as *Syngnathus* functioned as a catchall for any pipefish in the early paleontological literature.

Node 1: Stem Syngnathoidei
Fossil taxon: †*Gasterorhamphosus zuppichini* Sorbini 1981
Phylogenetic placement: MRCA of Syngnathoidei (Aulostomidae, Fistulariidae, Centriscidae (incl. *Macroramphosus*), Solenostomidae, Syngnathidae) and their sister group
Placement justification: The fossil was originally described as a Macroramphosidae [15] and later placed on the stem of Macroramphosidae+Centriscidae [13]. Macroramphosidae is now considered part of Centriscidae [16]. Several synapomorphies shared with a more inclusive group of Aulostomidae, Fistulariidae, Centriscidae, Solenostomidae and Syngnathidae: anal fin without spine; elongated tubular snout; pleural ribs absent; among others [109]. The grouping has previously been called Syngnathiformes (calibration 20 in [109] and calibration 14 [110]) but it has since been renamed to Syngnathoidei, while Syngnathiformes is now the more inclusive grouping also containing Dactylopteroidei (Dactylopteridae, Pegasidae), Mullidae and Callionymidae [16].
Stratigraphy: Upper Cretaceous "Calcari di Melissano", Porto Selvaggio, Lecce province, Italy
Minimum age: 83.6 Ma
Age justification: The fossil has originally been assigned a minimum age of 69.71 Ma [58], as the layers found at Nardò are dated to 70.08 Ma ± 0.37 Ma. Recently, it was suggested that the holotype may have been from a stratigraphically older locality [11]. Further, an unpublished fossil specimen from a stratigraphically lower position suggested a lower Campanian age [11], which is adopted here. We also investigated the importance of this fossil constraint on the ages of the other nodes by removing this calibration point and found only marginal differences.
Outgroup age sequence: 247.1, 236.0, 221.0, 193.81, 181.7, 166.1, 151.2, 150.94, 150.94, 125, 98, 98, 83.6
Estimated constraints on node age prior: mean 96.3 Ma, 95% CI 114.5 Ma.
Settings in BEAST: offsetlognormal(83.6,12.66,0.66)

Node 2: Stem Callionymidae
Fossil taxon: †*Gilmourella minuta* Carnevale & Bannikov 2019
Material: Description of holotype MCSNV T.381/T.382 [17].
Phylogenetic placement: MRCA of Callionymidae and their sister group
Placement justification: The fossil is placed as the sister group to Callionymidae, with Draconettidae as their sister group, based on five synapomorphies [17]: upper jaws strongly protractile; endopterygoid absent; hypural plate; haemal spine of the penultimate vertebra fused to the centrum; anal-fin rays mostly unbranched. Extant Draconettidae were shown with phylogenomic data to be the sister group to Callionymidae and their divergence was dated to ca. 70 Ma [11], which is consistent with a stem group placement of †*Gilmourella minuta*.
Stratigraphy: middle Eocene, Ypresian, Pesciara or Monte Bolca, Italy
Minimum age: 48.5 Ma
Age justification: The deposits from Monte Bolca's two principal Lagerstätten correspond to large parts of the Shallow Benthic Zone (SBZ) 11 of the late Ypresian Stage and have been dated to 48.5-50.7 Ma [14].

Note: To our knowledge, this is the first time this fossil was used in calibrating a time tree.
Outgroup age sequence: 247.1, 236.0, 221.0, 193.81, 181.7, 166.1, 151.2, 150.94, 150.94, 125, 98, 98, 83.6, 48.5
Estimated constraints on node age prior: mean 72.5 Ma, 95% CI 97.6 Ma.
Settings in BEAST: offsetlognormal(48.5,24.0,0.516)

Node 3: Stem Pegasidae
Fossil taxon: †*Ramphosus rosenkrantzi* Nielsen 1960
Material: Description of holotype NHMD 164232 [18]. Photos investigated: NHMD 624606 (DK 14), †*Ramphosus* sp. Danekræ fossil trove specimen, preserved as imprint in diatomite (part and counterpart). NHMD 625183 (DK 655), †*Ramphosus rosenkrantzi* Danekræ fossil trove specimen, preserved as imprint in diatomite (part and counterpart).
Phylogenetic placement: MRCA of Pegasidae and their sister group
Placement justification: The fossil and other members of †Ramphosidae show derived characteristics of Pegasidae [18]: elongated rostrum; ventral mouth opening; similar specialized jaw and head morphology; head covered in bony plates. There are also several differences, which separate †Ramphosidae from Pegasidae that place them on the stem of Pegasidae [13, 18, 46]: dorsal fin with single large spine in †Ramphosidae as opposed to to pterygial remnants in Pegasidae; trunk and tail covered by scales in †Ramphosidae compared to bony plates and rings in Pegasidae.
Stratigraphy: lower Eocene, Mo-Clay (Fur/Ølst) Formation, Denmark
Minimum age: 54 Ma
Age justification: The Mo-Clay of the Fur Formation bridges from the late Paleocene (NP9) into the early Ypresian (NP10) [20] and the layer was dated to 55.5-54.0 Ma, from which a minimum age of 54 Ma is derived.
Note: This fossil species is older than the †Ramphosidae †*Ramphosus rastrum* from Monte Bolca (48.5 Ma) used as the oldest known fossil by [11]. To our knowledge, this is the first time this fossil was used in calibrating a time tree.
Outgroup age sequence: 247.1, 236.0, 221.0, 193.81, 181.7, 166.1, 151.2, 150.94, 150.94, 125, 98, 98, 83.6, 54
Estimated constraints on node age prior: mean 75.3 Ma, 95% CI 98.4 Ma.
Settings in BEAST: offsetlognormal(54,21.3,0.532)

Node 4: Stem Aulostomidae + Fistulariidae
Fossil taxon: †*Eekaulostomus cuevasae* Cantalice & Alvarado-Ortega 2016
Material: Description of holotype IGM 4716 [21].
Phylogenetic placement: MRCA of Aulostomidae+Fistulariidae and their sister group
Placement justification: The fossil is the sister group of Aulostomidae+Fistulariidae based on phylogenetic analysis of 113 characters, of which 26 characters were assessed as being of high confidence [21]).
Stratigraphy: Paleocene, Danian, Tenejapa-Lacandón geological unit, Belisario Domínguez quarry, Salto de Agua Municipality, Chiapas, Mexico
Minimum age: 61.5 Ma
Age justification: The fossil was found in deposits of the Danian stage in a geological unit dated to 63 ± 1.5 Ma [21], and thus the minimum bound was set to 61.5 Ma.
Outgroup age sequence: 247.1, 236.0, 221.0, 193.81, 181.7, 166.1, 151.2, 150.94, 150.94, 125, 98, 98, 83.6, 61.5
Estimated constraints on node age prior: mean 75.3 Ma, 95% CI 98.4 Ma.
Settings in BEAST: offsetlognormal(61.5,17.5,0.562)

Node 5: Stem Centriscus+Aeoliscus
Fossil taxon: †*Gerpegezhus paviai* Bannikov & Carnevale 2012
Material: Description of holotype PIN 5314/1 [22]
Phylogenetic placement: MRCA of *Centriscus scutatus*+*Aeoliscus strigatus* and their sister group
Placement justification: Centriscidae has different systematic definitions, either separating *Macroramphosus*, *Notopogon* and *Centriscops* into Macroramphosidae as the sister group to a less inclusive Centriscidae (*Centiscus+Aeoliscus*) [13, 19], or including *Macroramphosus*, *Notopogon* and *Centriscops* within Centriscidae [16]. Both interpretations are consistent with the phylogeny recovered in this study and others [9, 11]. We use the wide definition of Centriscidae for the following discussion of the placement of †*Gerpegezhus paviai*.

The fossil was excluded by [11] because it was considered a stem Centriscidae and would thus be redundant with the calibration on Node 4 above. However, three or four synapomorphies place the fossil within Centriscidae as the sister group to *Centriscus+Aeoliscus* [22], separate from *Macroramphosus+Notopogon+Centriscops* (hereafter MNC): parietals present (absent in MNC, [13]); absence of upper procurrent caudal fin rays (MNC have five dorsal procurrent caudal fin rays, [13]); ventrolateral plates as a single row. We note that a fourth synapomorphy argued to unite the fossil and *Centriscus+Aeoliscus*, infraorbital series reduced to only lacrymal [22], could also be parsimoniously interpreted as an apomorphy of Centriscidae with a gain of a second infraorbital in *Centriscops*. Nonetheless, the remaining three apomorphies are considered strong evidence to include this fossil as a calibration point on the stem of *Centriscus+Aeoliscus*.
Stratigraphy: lower Eocene, Avazinka Formation, Kheu River, northern Caucasus, Russia
Minimum age: 55.728 Ma

Age justification: The stratigraphic layer bridges the late Paleocene (NP9) into the early Eocene (NP10) and has been dated to 55.964-55.728 Ma [22].
Note: The lower Eocene †*Protoramphosus parvulus* Danil'chenko 1968 is an equivalent fossil calibrating the same node based on the present sampling and is slightly younger (lower Eocene, Ypresian, Danatinsk Formation, Turkmenistan, 54.17 Ma [23]). The fossil can be placed on the stem of MNC based on elongate anterior vertebrae, the large dorsal spine, and the structure of the head [13]. To our knowledge, this is the first time this fossil was used in calibrating a time tree (see Placement justification).
Outgroup age sequence: 247.1, 236.0, 221.0, 193.81, 181.7, 166.1, 151.2, 150.94, 150.94, 125, 98, 98, 83.6, 61.5, 55.728
Estimated constraints on node age prior: mean 67.5 Ma, 95% CI 86.2 Ma.
Settings in BEAST: offsetlognormal(55.728,11.7,0.754)

Node 6: Stem Fistulariidae
Fossil taxon: †*Urosphenopsis sagitta* Danil'chenko 1968.
Material: Description of holotype PIN 2179/43
Phylogenetic placement: MRCA of Fistulariidae and their sister group.
Placement justification: This fossil has been recently placed in Aulostomoidea because the first four vertebrae are elongated and united, and ribs are absent [24]. The lack of squamation is shared with *Fistularia* [24], indicating a stem placement.
Stratigraphy: lower Eocene, Ypresian, Danatinsk Formation, Turkmenistan
Minimum age: 54.17 Ma
Age justification: Follows [24]. The deposits of the Danatinsk Formation of Turkmenistan calcareous nannoplankton zones NP9-NP10 [25], yielding a minimum age of 54.17 Ma [23].
Note: This fossil is older than the fossil †*Urosphen dubius* used as the oldest stem Fistulariidae by [11], which is dated to 48.5 Ma. To our knowledge, this is the first time this fossil was used in calibrating a time tree.
Outgroup age sequence: 247.1, 236.0, 221.0, 193.81, 181.7, 166.1, 151.2, 150.94, 150.94, 125, 98, 98, 83.6, 61.5, 54.17
Estimated constraints on node age prior: mean 66.7 Ma, 95% CI 85.9 Ma.
Settings in BEAST: offsetlognormal(54.17,12.51,0.725), with 'Use Originate' option

Node 7: Stem Solenostomidae
Fossil taxon: †*Solenorhynchus elegans* (Heckel 1853). Equivalent fossils from the same deposit are †*Solenorhynchus solenostomus* and †*Calamostoma lesiniforme* (Volta 1796), †*Calamostoma bolcensis* (Agassiz 1833).
Material: Description of type of †*Solenorhynchus elegans* [26].
Phylogenetic placement: Calibrated node is MRCA of Solenostomidae and their sister group

Placement justification: †*Calamostoma* and †*Solenorhynchus* are members of the extinct †Solenorhynchidae [26]. †Solenorhynchidae share five characteristic features with extant Solenostomidae: brood pouch formed by pelvic fins; body covered with stellate plates; pseudospines on dorsal and pelvic fins; pelvic fins have one pseudospine and six rays; no plostcleithrum [13, 26]. They are distinguished from crown Solenostomidae by pelvic fins that insert anterior of the spinous dorsal fin [26], placing them on the stem of Solenostomidae.
Stratigraphy: middle Eocene, Ypresian, Pesciara or Monte Bolca, Italy
Minimum age: 48.5 Ma
Age justification: The deposits from Monte Bolca's two principal Lagerstätten correspond to large parts of the Shallow Benthic Zone (SBZ) 11 of the late Ypresian Stage and have been dated to 50.7-48.5 Ma [14].
Outgroup age sequence: 247.1, 236.0, 221.0, 193.81, 181.7, 166.1, 151.2, 150.94, 150.94, 125, 98, 98, 83.6, 48.5
Estimated constraints on node age prior: mean 72.5 Ma, 95% CI 97.6 Ma.
Settings in BEAST: offsetlognormal(48.5,24.0,0.516)

Node 8: Stem Microphini+Doryramphini+Maroubra+Heraldia (spiny ridge clade)
Fossil taxon: †*Maroubrichthys serratus* Parin 1992
Material: Description of holotype PIN No. 3363-30 [27]
Phylogenetic placement: MRCA of *Maroubra*, *Heraldia*, Microphini and Doryramphini and their sister group
Placement justification: The fossil has a brood area under the trunk [27], placing it in Nerophinae. It has spiny ridges with denticulate margins on the body rings, which is characteristic of members of the clade Doryrhamphini+Microphini+*Heraldia*+*Maroubra* (Table S2). Their sister group Nerophini have more smooth bodies without denticulate margins on their body rings. The fossil also has a longitudinal opercular ridge [27], which is also present in members of the spiny ridge clade except for some species of *Microphis* [43], while Nerophini lack opercular ridges in adults, albeit present in juveniles of *Leptoichthys* [43]. The original description of the fossil argued for an affiliation with *Maroubra* [27] but the remaining listed characters are not apomorphic for *Maroubra* or for others within the spiny ridge group (Table S2): moderate elevation of dorsal medial snout ridge without spines or denticles (also present in Microphini), a caudal fin with 10 rays (also present in Doryramphini and in *Choeroichthys* in Microphini), pouch plates covering the brood pouch (not present in *Maroubra*). We therefore argue for a stem group placement on the spiny ridge clade based on the apparently apomorphic spiny body ridges and opercular ridges.
Stratigraphy: lower Oligocene, Rupelian, Pshekhskiy horizon of the lower Maikop series, Adygeya, Belaya River above Abadzekhskaya, Caucasus [27].
Minimum age: 32.25 Ma
Age justification: The Pshekhzskiy horizon lies between the base of the Paleogene Nannoplankton zone NP23, which has been dated to 32.25 Ma (no dating error specified in [28]), and Zone P18 of the

Paleogene planktic foram zonation scheme, which has been dated to 33.9±0.1 [28] and references therein), yielding a minimum age of 32.25 Ma.
Note: †*Pshekhagnathus polypterus* Bannikov et al. 2017 (holotype, PIN 5419/2, likely also *Acanthognathus* (= *Doryrhamphus*) *squalidus* Danil'chenko 1960 [70, 29]) from the same stratum and locality may calibrate the same node as †*Maroubrichthys serratus* but it is less completely preserved. We discuss some points that may help in a future placement. The fossil has a fully developed anal fin [70] unlike all extant syngnathids that have minute anal fins. The position of the brooding area is unknown [70], which leaves the possibility that the species belongs to Syngnathinae. On the other hand, the fossil has spines on rings and a longitudinal opercular ridge like members of the Nerophinae spiny ridge clade and †*Maroubrichthys serratus*. To our knowledge, this is the first time this fossil was used in calibrating a time tree.
Outgroup age sequence: 247.1, 236.0, 221.0, 193.81, 181.7, 166.1, 151.2, 150.94, 150.94, 125, 98, 98, 83.6, 48.5, 32.25
Estimated constraints on node age prior: mean 52.5 Ma, 95% CI 80.5 Ma.
Settings in BEAST: offsetlognormal(32.25,20.21,0.662)

Node 9: Stem Microphini+Doryrhamphini
Fossil taxon: †*Doryrhamphus* sp. Micklich & Parin 1996
Phylogenetic placement: calibrated node is MRCA of Microphini+Doryrhamphini and their sister group
Placement justification: The fossil has a brood area under the trunk [30] and is therefore a Nerophinae. It can be further assigned to the clade of Doryramphini+Microphini+*Heraldia*+*Maroubra* due to its spines distally on the body rings and the presence of a longitudinal opercular ridge (Table S2). Additional characters that are preserved are not apomorphic for more inclusive clades: the prominent caudal fin is found in Doryrhamphini, Microphini and *Heraldia* but absent in *Maroubra*; the number of rings is overlapping with most groups (40-44 body rings, 15-17 trunk rings, [30], (Table S2). The fossil has an elongated pectoral ring, which is characteristic of Doryrhamphini, and keeled scutella, which are characteristic of Microphini. Given this occurrence of both keeled scutella and the hypertrophied pectoral ring in the same species, these traits may not be apomorphic of the crown groups but of Microphini+Doryrhamphini, with subsequent loss in one of these groups. We therefore place the fossil on the stem of Microphini+Doryrhamphini.
Stratigraphy: early Oligocene, Rupelian, Frauenweiler, part of the Rauenberg clay pits, Germany
Minimum age: 29.5 Ma
Age justification: The Frauenweiler fossil site is part of the Rauenberg clay pits, which have been placed in nannoplankton zone NP23, dinoflagellate based subzone D14a and mammalian creodont based zone MP22-MP23, leading to a stratigraphic range of 29.5-32 Ma [31].
Note: Possible equivalent fossils also from the Rupelian but from different geographic settings are: †*Doryrhamphus fredericae* (Pharisat 1991), France; †*Doryrhamphus incolumis* (Danil'chenko 1960),

Lower Khadum Formation, Lower Maikop group, North Caucasus; †*Microphis* sp., Frauenweiler, Germany. To our knowledge, this is the first time this fossil was used in calibrating a time tree.
Outgroup age sequence: 247.1, 236.0, 221.0, 193.81, 181.7, 166.1, 151.2, 150.94, 150.94, 125, 98, 98, 83.6, 48.5, 48.5, 32.25, 29.5
Estimated constraints on node age prior: mean 38.1 Ma, 95% CI 53.9 Ma.
Settings in BEAST: offsetlognormal(29.5,8.6,0.86)

Node 10: Stem Nerophis
Fossil taxon: †*Hipposyngnathus neriticus* Jerzmańska 1968
Material: Description of holotype A/533 [32]; Pl; Pi-F/MP/4a/1572/10 (counterpart Pi-F/MP/4b/1572/10), skull and pre-anal part of the body, with proximal parts of several dorsal fin rays; Pi-F/MP/5a/1572/10 (counterpart Pi-F/MP/5b/1572/10) abdomen [33].
Phylogenetic placement: MRCA of *Nerophis* (including *Entelurus*) and their sister group
Placement justification: The fossil is a trunk brooder, likely placing it in Nerophinae, and lacks denticulate margins and lacks an opercular ridge [32, 33], which excludes the clade characterized by spiny ridges. Within Nerophini, it shares continuous superior ridges with *Nerophis+Entelurus*, which are discontinuous in *Leptoichthys fistularius* (Table S2), which places the fossil on the stem of *Nerophis+Entelurus*.
Stratigraphy: lower Oligocene, Rupelian, Hermanowa locality, Poland
Minimum age: 30.6 Ma
Age justification: The species in found in the lowermost part of the profile (H5 layer) from the Hermanowa locality in the NP23 calcareous nannoplankton zone, equivalent to the IPM2 ichthyofaunal zone, which ranges from 30.6-32.3 Ma [34].
Note: To our knowledge, this is the first time this fossil was used in calibrating a time tree.
Outgroup age sequence: 247.1, 236.0, 221.0, 193.81, 181.7, 166.1, 151.2, 150.94, 150.94, 125, 98, 98, 83.6, 48.5, 48.5, 32.25, 30.6
Estimated constraints on node age prior: mean 38.7 Ma, 95% CI 54.0 Ma.
Settings in BEAST: offsetlognormal(30.6,8.1,0.88)

Node 11: Stem pygmy pipehorses
Fossil taxon: †*Hippotropiscis frenki* Žalohar & Hitij 2012
Material: description of holotype [47]
Phylogenetic placement: MRCA of *Cylix tupareomanaia+Acentronura tentaculata* and their sister group
Placement justification: The fossil was described as an intermediate between *Idiotropiscis* (a pygmy pipehorse) and *Hippocampus* (seahorses), being morphologically closer to seahorses than to the pygmy pipehorses [47]. This interpretation may have been guided by a previous phylogeny that showed pygmy pipehorses as sister to seahorses [52]. Taxonomically denser phylogenies showed a more complicated

relationship with several pipefish lineages that are more closely related to the seahorses and "pygmy pipehorses" than they are to each other ([44]; this study). These new findings require a reinvestigation of the affiliation of †*Hippotropiscis frenki*. The thorough morphological description by [47] allows for a placement on the stem pygmy pipehorses (*Idiotropiscis* including *Acentronura tentaculata*) rather than with seahorses. Like pygmy pipehorses the fossil has pouch plates (absent in seahorses) and an obtuse angle of the head to the body (head in a sharp angle to the trunk in seahorses). Within pygmy pipehorses, the fossil shares the discontinuous tail and trunk ridges with members of *Idiotropiscis* but not with *Acentronura*. Given the inclusion of *Acentronura* within *Idiotropiscis*, the discontinuous tail and trunk ridges could be a stem trait of pygmy pipehorses, with a loss in *Acentronura*. The elevated frontal ridge of the fossil and *Idiotropiscis lumnitzeri* and *I. australe*, but not *Acentronura* or *Cylix*, suggests at least two gains given the present phylogeny and is therefore not useful in placing the fossil.
Stratigraphy: middle Miocene, lower Sarmatian, Coprolithic horizon, Tunjice Hills, Slovenia
Minimum age: 11.6 Ma
Age justification: The Sarmatian stage in the area covered a time span between 12.7-11.6 Ma [35], leading to a hard minimum age of 11.6 Ma.
Note: To our knowledge, this is the first time this fossil was used in calibrating a time tree (it was only used to inform the prior distribution for the seahorse divergence [49]).
Outgroup age sequence: 247.1, 236.0, 221.0, 193.81, 181.7, 166.1, 151.2, 150.94, 150.94, 125, 98, 98, 83.6, 48.5, 48.5, 32.25, 11.6
Estimated constraints on node age prior: mean 29.2 Ma, 95% CI 50.5 Ma.
Settings in BEAST: offsetlognormal(11.6,17.6,0.586)

Node 12: Stem Haliichthys+Halicampus grayi+Trachyrhamphus+Filicampus
Fossil taxon: †*Hippohaliichthys edis* Žalohar & Hitij 2017
Material: description of holotype [48]
Phylogenetic placement: *Haliichthys+Halicampus grayi+Trachyrhamphus+Filicampus* and their sister group
Placement justification: Upon its description, this fossil was interpreted as an intermediate between *Haliichthys* and seahorses [48]. *Haliichthys taeniophorus* is more closely related to several pipefish lineages in Haliichthyini (*Halicampus grayi, Filicampus tigris*, species of *Trachyrhamphus*) than to seahorses. The fossil and members of the clade within Haliichthyini share discontinuous superior trunk and tail ridges, while the sister group *Lissocampus* has continuous superior ridges [44].
Stratigraphy: middle Miocene, lower Sarmatian, Coprolithic horizon, Tunjice Hills, Slovenia
Minimum age: 11.6 Ma
Age justification: The Sarmatian stage in the area covered a time span between 11.6-12.7 Ma [35].
Note: To our knowledge, this is the first time this fossil was used in calibrating a time tree (it was only used to inform the prior distribution for the seahorse divergence [49]).
Outgroup age sequence: 247.1, 236.0, 221.0, 193.81, 181.7, 166.1, 151.2, 150.94, 150.94, 125, 98, 98, 83.6, 48.5, 48.5, 32.25, 11.6, 11.6
Estimated constraints on node age prior: mean 20.6 Ma, 95% CI 37.8 Ma.

Settings in BEAST: offsetlognormal(11.6,9.0,0.99)

Node 13: Crown Hippocampus
Fossil taxa: †*Hippocampus slovenicus* Žalohar et al. 2009
Material: description of holotype [35]
Phylogenetic placement: MRCA of *Hippocampus* including pygmy seahorses and seahorses
Placement justification: The fossil is similar to extant seahorses [35], placing them in the crown group. †*Hippocampus slovenicus* was suggested to be a relative of pygmy seahorses [35]. The distinction between pygmy seahorses and non-pygmy seahorses includes a number of characteristics (in addition to their phylogenetic distinctness): Pygmy seahorses are diminutive in body size, have less tail rings, brood on the trunk, and have a smaller number of dorsal and pectoral fin rays compared to other seahorses [36]. The fossil †*Hippocampus slovenicus* does have a low number of tail rings (25-26) similar to other pygmy seahorses (range 26-30 rings). However, it has 15-16 dorsal fin rays, which is higher than in extant pygmy seahorses (range 7-14) and more consistent with the range of the other non-pygmy seahorses (range 11-33). As their names implies, pygmy seahorses also have a diminutive body size and the fossils are tiny as well (6-15 mm). However, body size reduction also happened within the non-pygmy clade of seahorses (e.g. *H. zosterae*). The interpretation of †*Hippocampus slovenicus* as a crown pygmy seahorse is therefore uncertain. A stem position seems more appropriate given the shared similarities with non-pygmy seahorses, which effectively places the fossil on the crown of all *Hippocampus* in node dating.
Stratigraphy: middle Miocene, lower Sarmatian, Coprolithic horizon, Tunjice Hills, Slovenia
Minimum age: 11.6 Ma
Age justification: The Sarmatian stage in the area covered a time span between 11.6-12.7 Ma [35], which serves as the minimum age used here.
Outgroup age sequence: 247.1, 236.0, 221.0, 193.81, 181.7, 166.1, 151.2, 150.94, 150.94, 125, 98, 98, 83.6, 48.5, 48.5, 32.25, 11.6, 11.6
Estimated constraints on node age prior: mean 20.6 Ma, 95% CI 37.8 Ma.
Settings in BEAST: offsetlognormal(11.6,9.0,0.89)

Node 14: Crown non-pygmy Hippocampus excluding H. jugumus
Fossil taxa: †*Hippocampus sarmaticus* Žalohar et al. 2009
Material: description of holotype [35]
Phylogenetic placement: MRCA of *Hippocampus abdominalis* and *Hippocampus reidi*
Placement justification: †*Hippocampus sarmaticus* was described as a close relative to the extant *H. trimaculatus* [35] and adopted for fossil calibration of the seahorse phylogeny [49] with strong impacts on the age estimates as discussed in the main text. However, this affiliation is in our view difficult given that the putatively shared characteristics between the fossil and *H. trimaculatus* show high plasticity across *Hippocampus* and are shared with other seahorse species: head to snout length ratio 1.8-2.5

(shared with at least 23 seahorse species), a relatively high number of 42-43 tail rings (overlapping with *H. trimaculatus*: 38-43 but also with *H. breviceps*: 39-43 and *H. dahli*: 40-42 [51]), 11 trunk rings (shared with at least 26 species), one cheek spine (shared with 28 species [51]), the dorsal fin supported by 2 trunk and 1 tail ring (shared with at least 25 species), and the narrow head with low coronet in line with the arc of the neck (low coronets are shared by 15 species [51]). While the fossil clearly appears to be a member of the crown of non-pygmy seahorses due to these shared characteristics, none of the characters are diagnostic to place it among an extant non-pygmy seahorse clade.

The fossil shares 11 trunk rings with most non-pygmy seahorses (range 11-12), distinct from the sister group *H. jugumus* (13 trunk rings). We therefore place the fossil on the crown of non-pygmy seahorses excluding *H. jugumus*.

The fossil was previously used on the crown group of *Hippocampus*, defined by the sampling as the MRCA of *H. abdominalis* and other seahorses [11]. Therefore, the placement of the fossil was the same as here, although we argue that it is not a crown member of *Hippocampus* in its entirety. Here, by including pygmy seahorses and *H. jugumus*, we show that the crown group of *Hippocampus* is larger.
Stratigraphy: middle Miocene, lower Sarmatian, Coprolithic horizon, Tunjice Hills, Slovenia
Minimum age: 11.6 Ma
Age justification: The Sarmatian stage in the area covered a time span between 11.6-12.7 Ma [35], which serves as the minimum age used here.
Outgroup age sequence: 247.1, 236.0, 221.0, 193.81, 181.7, 166.1, 151.2, 150.94, 150.94, 125, 98, 98, 83.6, 48.5, 48.5, 32.25, 11.6, 11.6, 11.6
Estimated constraints on node age prior: mean 16.2 Ma, 95% CI 28.1 Ma.
Settings in BEAST: offsetlognormal(11.6,4.6,1.25)

Fossil taxon only used in biogeographic analysis

In addition to the 14 fossils used for fossil calibration, we used †*Protoramphosus parvulus* (Node 5b in Fig. 5a), which was not used for node calibration because it calibrated the same node as another fossil (Node 5, MRCA of Centriscidae), but could inform the biogeographic reconstruction applied to the lineage leading to *Macroramphosus*.

Systematics

Syngnathidae Bonaparte 1831 are divided into the subfamilies Nerophinae Kaup 1853 and Syngnathinae Bonaparte 1831. Further clades within the subfamilies were identified [44] but not named. In order to facilitate communication about the deeply diverged and diverse clades, we are establishing names as zoological tribes within both Nerophinae and Syngnathinae using available names where possible. New tribes have been registered under ZooBank (urn:lsid:zoobank.org:pub:3D1F608C-B8B0-4C8E-B2DD-E0F88D73E09B). We list all contained genera as currently known and characteristics.

Tribes in Nerophinae

Nerophini Kaup 1853. Contains *Entelurus*, *Nerophis* and the brushtail pipefish *Leptoichthys fistularius*. We find *Nerophis* to include the monotypic *Entelurus aequoreus*. Both groups occur in European waters, while the snake pipefish is even distributed in Icelandic waters. *Leptoichthys fistularius* is monotypic and occurs in temperate Australian waters. Temperate Australasia and northeastern Atlantic.

Doryrhamphini Kaup 1856. Contains the flagtail pipefishes *Doryrhamphus* and *Dunckerocampus*. Note that some authors consider *Dunckerocampus* a subgenus of *Doryrhamphus* [43]. The two groups differ in their pouch structure (absence of pouch folds in species of *Dunckerocampus*; presence in *Doryrhamphus*). We recover them as reciprocally monophyletic and the well separated groups. Indo-Pacific distribution.

Microphini new tribe
Type genus: *Microphis* Kaup 1853.
Diagnosis: Superior trunk and tail ridges continuous (*Choeroichthys*) or discontinuous (*Doryichthys* + *Microphis*), lateral trunk ridge confluent with inferior tail ridge (*Choeroichthys* + *Microphis*) or typically straight and ends near anal ring (*Doryichthys*), inferior trunk ridge ends on anal ring (*Choeroichthys* + *Microphis*) or continuous with tail ridge (*Doryichthys*), median dorsal snout ridge low, entire, dorsal-fin origin on trunk, brood pouch under trunk, pouch plates present, pouch folds present (*Choeroichthys*) or absent (*Doryichthys* + *Microphis*). Atlantic and Indo-Pacific.

Contains *Choeroichthys*, *Microphis* and *Doryichthys*. Our extended sampling shows *Doryichthys* Kaup 1856 as deeply nested within *Microphis* Kaup 1853, making *Doryichthys* a junior synonym of

Microphis. Both groups are distributed mostly in the tropical Indo-Pacific and are morphologically similar. At least the subadults and adults inhabit fresh and estuarine waters. The genera are distinguished by discontinuous inferior trunk and tail ridges (*Doryichthys*) or discontinuous ridges (*Microphis*).

Tribes in Syngnathinae

Solegnathini Gill 1859. Clade number ii in [44]. Syngnathoidinae Fowler 1951 as Syngnathoidini and Phyllopteryginae Fowler 1951 as Phyllopterygini are available but can be treated as junior synonyms of Solegnathini (based on Fig. 2b). Contains *Solegnathus*, *Syngnathoides*, *Phycodurus* and *Phyllopteryx*. Note that [37] referred to *Solegnathus* as *Solenognathina*, but this name cannot be accepted as it was based on a now invalid name *Solenognathus* Agassiz, 1846. The monotypic alligator pipefish *Syngnathoides biaculeatus* is one of two examples of tail brooders that have secondarily moved the placement of their eggs onto the trunk [46]. The seadragons are known for their camouflage: The leafy and common seadragon have dermal appendages imitating kelp leaves, while the ruby seadragon is missing the appendages [38]. Indo-Pacific distribution.

Stigmatoporini new tribe
Type genus: *Stigmatopora* Kaup 1853.
Diagnosis: Superior and inferior trunk ridges continuous with their respective tail ridges (*Stigmatopora*) or discontinuous (*Corythoichthys*), lateral trunk ridge ends mid laterally between the penultimate trunk ring and 35th tail ring (*Stigmatopora*) or ends near anal ring (*Corythoichthys*), inferior trunk and tail ridges continuous (*Corythoichthys*), median dorsal snout ridge low, entire, dorsal-fin origin on the trunk ring (*Stigmatopora*) or between anterior margin of last trunk ring and posterior margin of 1st tail ring (*Corythoichthys*), brood pouch under the tail, pouch plates absent, pouch folds present, and semi pouch-closure. *Stigmatopora* possesses a slender distally coiled prehensile tail. Dimorphic coloration under head and 1st-2nd trunk rings. Indo-Pacific.

Clade number i in [44]. Contains *Stigmatopora* and *Corythoichthys*. *Stigmatopora* is a south Australian and New Zealand endemic group. *Corythoichthys* species are distributed throughout the Indo-Pacific and show remarkable color camouflage patterns to mimic the corals they live on.

Leptonotini new tribe

Type genus: *Leptonotus* Kaup 1853.
Diagnosis: A large grouping of morphologically disparate genera of pipefish that appear difficult to be united by broad diagnostic characters, including (1) the worm-like genera *Apterygocampus*, *Campichthys, Nannocampus* and *Siokunichthys*; (2) *Bhanotia* and the euryhaline *Hippichthys;* (3) the Australasian temperate genera *Pugnaso*, *Vanacampus, Histiogamphelus, Leptonotus, Kaupus, Mitotichthys* and *Hypselognathus*; (4) *Cosmocampus* (Pacific), *Festucalex*, and *Phoxocampus*; (5) the prehensile *Urocampus;* and (6) *Micrognathus* and *Minyichthys*. Indo-Pacific distribution.

Clade number iv in [44]. Contains two main clades, one of *Apterygocampus*, *Bhanotia, Campichthys, Nannocampus*, *Hippichthys* including *Ichthyocampus*, and *Siokunichthys*; the other of *Cosmocampus* (in part: Pacific clade) including *Phoxocampus*, *Histiogamphelus, Hypselognathus, Festucalex*, *Kaupus, Leptonotus, Micrognathus*, *Minyichthys*, *Mitotichthys* (non-monophyletic), *Pugnaso*, *Urocampus,* and *Vanacampus*. The type genus of Leptonotini, *Leptonotus*, which was included in a phylogeny for the first time, is part of a clade of 7 genera all endemic to temperate Australia and New Zealand (*Hypselognathus, Kaupus, Leptonotus, Histiogamphelus, Mitotichthys, Pugnaso, Vanacampus*). This large group contains groups of various morphologies and life styles. *Hippichthys* is distributed in estuarine systems of the Indo-Pacific, some species living in freshwater. *Hippichthys* Bleeker 1849 was found here to include the Indian freshwater pipefish *Ichthyocampus carce* Kaup 1853, and *Ichthyocampus* is therefore considered a junior synonym. Further non-monophyly was found in *Festucalex*, *Mitotichthys* and *Cosmocampus*. Because the type species *C. albirostris* is in Syngnathini (see below), the five "*Cosmocampus*" species in Leptonotini will have to be included into other existent genera or new genera.

<u>Syngnathini</u> Bonaparte 1831. Clade number v in [44]. Contains *Amphelikturus, Anarchopterus, Cosmocampus* (in part, Atlantic clade) including *Bryx*, *Enneacampus, Halicampus* (in part: Atlantic *Halicampus* and short-snouted *Halicampus*), *Penetopteryx, Pseudophallus,* and *Syngnathus*. Syngnathini contains the speciose genus *Syngnathus*, which contains many species that are the "model species" of Syngnathidae and are being studied for their sex role reversal and variable mating patterns. Globally distributed.

This large group contains one of three occurrences of members of *Halicampus* recovered in our tree. Based on morphological observations, [43] suggested that *Halicampus* could be paraphyletic in three groups but kept the genus nonetheless. Two of the groups were found based on molecular data [44]. The placement of the short snouted *Halicampus* is strongly supported as the sister group to all

other Syngnathini, while the placement in [44] had no support. The species of *Halicampus* in Syngnathini all have short snouts [43], pouches with pouch plates and a semi-closed pouch closure. If the short snout proves to define this group, we can predict that the following species may fall in this clade: *H. spinirostris* (Dawson & Allen 1981), *H. zavorensis* Dawson 1984 and *H. edmondsoni* Pietschmann 1928. No genus names are available for this group, and if a new genus would be erected, a new name would be necessary.

Syngnathini contains the second group of *Cosmocampus*, including the type species *C. albirostris*. This group is found on both sides of the Isthmus of Panama, and includes *Bryx randalli*. More sampling of *Bryx* species is needed to assess the potential synonymy including the type species *B. veleronis* Herald 1940.

<u>Haliichthyini</u> Whitley & Allan 1958. Part of clade number vi in [44]. Acentronurinae Whitley & Allan 1958 as Acentronurini is available and a synonym. We prefer Haliichthyini on the basis of prevailing usage in [43]. Contains *Trachyrhamphus, Filicampus, Halicampus grayi, Haliichthys, Lissocampus, Idiotropiscis,* and *Acentronura*. This group contains a number of morphologically distinct syngnathids, some "classic" pipefishes but also the Pacific lineage of "pygmy pipehorses" (*Cylix*, *Idiotropiscis, Acentronura*). Haliichthyini includes the type species of *Halicampus*, which has not been included in a previous phylogeny. Indo-Pacific distribution.

<u>Hippocampini</u> Bonaparte 1835. Clade number vii and part of clade number vi in [44]. The clade is amended here to contain the long-snouted *Halicampus* (*H. macrorhynchus* and *H. punctatus*) and pygmy and non-pygmy seahorses *Hippocampus*. This is the third occurrence of *Halicampus* on the tree and contains species with long snouts (1.5-1.9 in head length [43]). [39] mention that *H. macrorhynchus* has an unprotected brood pouch with only pouch folds, while members of the short-snouted group have both pouch plates and pouch folds [43]. Available names are *Phanerotokeus gohari* Duncker 1940 which was synonymized with *Halicampus macrorhynchus*, and which precedes *Yozia punctata* Kamohara 1952. Globally distributed.

<u>*Stipecampus cristatus*</u>. Clade number iii in [44]. Contains a single species, the ring-back pipefish. Endemic to southwestern Australia.

Taxonomy

Our sampling identified (1) potential new species, (2) species in previous synonymy, (3) non-monophyletic genera (including implications for synonymous genus names), (4) extended biogeographic realms. We list the current evidence below and recommend taxonomic actions if sufficient evidence exists.

(1) Potential new species

A) *Doryrhamphus excisus* complex: This lineage was shown to have at least five cryptic species based on mitochondrial DNA [60] and public barcode data suggests that it could be at least eight. We sampled nine specimens of the *D. excisus* complex (of the putative species *D. excisus*, *D. japonicus, D. melanopleura, D. californiensis* and *D. paulus*). We find specimens of *D. excisus* from the Central Pacific and the Central Indopacific are forming three putative species. Additionally we find 3 species for which names are available, which could be removed from the synonymy with *D. excisus* (see next section "(2) Synonymized species"). Resolving the synonyms of *Doryrhamphus excisus* Kaup 1856 will be a difficult task because the type locality is unknown [40]. The three putative new species in the complex are:

A1) *Doryrhamphus excisus* sp1. This putative species has been sampled in the Seychelles and Marquesas Islands. Given the short branch lengths separating the two specimens, the putative species is likely distributed at least across the Central Indopacific areas that lie between the two sampling localities. Comparison to publicly available COI barcodes also shows two other specimens from the Seychelles (99.4% identity, SAIAB 78058-T455, SAIAB 78058-T456) from the same lot as our Seychelles specimen (SAIAB 78058), while other *Doryrhamphus* species are at least 8% divergent.

A2) *Doryrhamphus excisus* sp2. This putative species has been sampled from the Philippines. Comparison to publicly available COI barcodes shows this species is also present in Lizard Island, Australia (99.4% identity, LIFSA041-08), with the caveat that the obtained COI fragment from our specimen is short (292 bp).

A3) *Doryrhamphus excisus* sp3. This putative species is represented by a specimen from Guam and the Northern Mariana Islands. The two specimens have a COI pairwise distance in available COI barcodes of at least 6.5% to other *Doryrhamphus* species.

B) *Halicampus dunckeri* is sampled with two specimens from the Philippines here, which are sister groups but are separated by long branches. Without material from the type locality in Indonesia, it cannot be discerned which lineage represents the nominal *H. dunckeri*.

C) *Hippichthys penicillus* is represented by two specimens from Kuwait separated by long branches, while one of the lineages is also represented by a specimen from Australia. Without material from the type locality in Malaysia, it cannot be discerned which lineage represents the nominal *H. penicillus*.

D) *Hippocampus mohnikei* sp2 was suggested as potentially cryptic based on barcoding data [51], and we also find a substantial divergence in nuclear DNA. Publicly available COI barcodes show that our specimen from Malaysia is >99% identical in sequence to specimens from India (GenBank accession numbers MN595217, MK330041, MN595216). The occurrence of *H. mohnikei* in Indian waters was interpreted as a range expansion of *H. mohnikei* [62] but with a minimum divergence in COI of 7.8% from nominal *H. mohnikei*, the interpretation as a separate species may be more justified.

E) *Stigmatopora nigra* sp2. *Stigmatopora* was shown to have strong genetic structure across its range, possibly warranting description of 2 new species, in addition to the nominal *S. nigra* [61]. We sampled one of these undescribed species from New Zealand (*S. nigra* sp2 in the main figures which corresponds to "*Stigmatopora_nigra2*" in [61]) and confirmed some genetic distance to the nominal *S. nigra* (here sampled from Queensland, Australia).

(2) Synonymized species that are distinct according to the phylogenetic hypothesis

A) *Doryrhamphus melanopleura* (Bleeker 1858) is treated as a synonym of *D. japonicus* Araga & Yoshino 1975 by [12]. We find this species from the Society Islands and *D. japonicus* in different clades, arguing for the validity of *D. melanopleura*. Comparison to publicly available COI barcodes shows various specimens from localities across French Polynesia including Society Islands (99-100% identity).

B) *Doryrhamphus californiensis* Gill 1863 (type locality: Cape St. Lucas, Baja California, Mexico) is an existing name that is considered a synonym of *Doryrhamphus excisus* [43] . Our specimen is from the Gulf of California and is separated from another specimen from the Gulf of California by long branches, and in a different clade from other specimens of *D. excisus*. We therefore tentatively resurrect the synonym for this species.

C) *Doryrhamphus paulus* Fritzsche 1980 (type locality: Isla Socorro, Revilla Gigedo Islands, off western Mexico) is an existing name that is considered a synonym of *Doryrhamphus excisus* [40] or a subspecies of *D. excisus* [43]. Our specimen is from the Gulf of California and is separated from another specimen from the Gulf of California (tentatively *D. californiensis*, see above) by long branches, and in a different clade from other specimens of *D. excisus*. We therefore tentatively resurrect the synonym for this species.

D) *Hippocampus borboniensis* Dumeril 1870 is treated as a synonym of *H. kuda* Bleeker, 1852 [51]; however these species are shown here to be in separate clades within. *Hippocampus borboniensis* is thus considered valid. The status of *H. borboniensis* is however not completely clear because the two specimens from Tanzania and Madagascar were also including *H. capensis* Boulenger, 1900. *Hippocampus capensis* is ecologically, geographically and morphologically distinct, and is considered Endangered by the IUCN. The species in the *H. kuda* complex are closely related and difficult to resolve due to their rapid divergence.

E) *Solenostomus paegnius* Jordan & Thompson 1914 was considered a synonym of *S. cyanopterus* [41], whereas others [40] treat it as a separate species. The phylogeny recovered *S. paegnius* as more closely related to *S. paradoxus*, both of which are separated by long branches, therefore supporting a separate species status.

(3) Non-monophyletic genera

A) *Cosmocampus* Dawson 1979 occurs in two places in the tree, one group that is largely distributed in the Atlantic Ocean with the exception of *C. arctus* in the eastern Pacific and one clade in the Pacific. *Cosmocampus albirostris* (Kaup 1856) in the Atlantic clade is the type species (type locality: Mexico; our specimen is from Cozumel, Mexico), which implies that the Pacific group will require a new genus

name. Because the Pacific "*Cosmocampus*" also includes *Phoxocampus* Dawson 1977, an option would be to broaden *Phoxocampus* to include Pacific *Cosmocampus*.

B) *Festucalex* Whitley 1931 occurs in two places of the tree, one clade of *F. cinctus* (Ramsay 1882) and *F. scalaris* (Günther 1870), the other of *F. erythraeus* (Gilbert 1905). *Festucalex cinctus* (Ramsay 1882) is the type species by original designation and was described from Port Jackson, New South Wales. Our specimen of *F. cinctus* is from the close-by Nelson Bay, New South Wales. We therefore suggest that *Festucalex* remains with the clade of *F. cinctus* and *F. scalaris*, and that *F. erythraeus* is placed in a new genus.

C) *Halicampus* Kaup 1856 occurs in four places of the tree. Morphological examination of the type species *H. grayi* Kaup 1856, the long-snouted forms (*H. macrorhynchus*, *H. punctatus*) and the short-snouted forms were already suggested as three separate lineages based on morphological examination but the evidence was considered too preliminary to change the systematics [43]. The fourth lineage is *H. crinitus* in the Atlantic, which was either treated as members of *Micrognathus* [43] (which would then also make *Micrognathus* paraphyletic according to our data which supports *Micrognathus* as part of Leptonotini) or as members of *Halicampus* [40]. Our genetic data confirms these four predicted groups of *Halicampus*.

Halicampus grayi is the only *Halicampus* with 17−18 trunk rings, while all other species have only 13−15 trunk rings. No pouch plates but pouch folds are present. The name *Halicampus* will remain with the type species *H. grayi*, while the other three clades need to be placed in new genera. The type locality of *Halicampus grayi* was described with a vague type locality "India or Australia". Our specimens of *H. grayi* are from northern Australia and Japan. For the long-snouted clade, possible available genera names include *Yozia* for *Yozia punctata* Kamohara 1952, *Phanerotokeus* for *Phanerotokeus macrorhynchus* (Duncker 1940) and *Halicampoides* Fowler, 1956 for *Halicampoides macrorhynchus*. For the short-snouted clade, there are no available genus names that could be resurrected. Within the short-snouted species, two distinct lineages were found in our analysis that show morphological differences associated with the snout, based on the presence of a continuous or discontinuous median dorsal ridge. Whether this distinction holds up should be confirmed by sampling other short snouted species that have not been sampled here (*H. edmondsoni, H. spinirostris, H. zavorensis*).

D) *Hippichthys* Bleeker 1849 is shown to include the monotypic *Ichthyocampus carce* (Hamilton Buchanan 1822). *Ichthyocampus* Kaup 1853 is therefore suggested as a junior synonym of *Hippichthys*. The simplest taxonomic change is to include the species in *Hippichthys*.

E) *Idiotropiscis* Whitley 1947 is shown to include *Acentronura tentaculata* Günther 1870. We currently do not know if the type species *A. gracilissimus* Temminck & Schlegel 1850 also falls within *Idiotropiscis*, which is required to recommend on nomenclatural changes in the group.

F) *Microphis* Kaup 1853 is shown to include *Doryichthys* Kaup 1856, confirming findings based on fewer taxa [9, 11]. The type species *M. cuncalus* falls in one clade that is the sister to *Doryichthys* and several *Microphis* species. Both an extension of *Microphis* to include *Doryichthys* species is possible or a new genus name for the second clade (containing *M. aculeatus, M. lineatus, M. brachyurus, M. jagorii* and *M. brevidorsalis*).

G) *Mitotichthys* Whitley 1948 appears twice in the tree. We did not sample the type species *M. tuckeri* (Scott 1942). The generic definition was largely based on *M. tuckeri* and *M. semistriatus* [43], and we therefore predict that the type species may group with *M. semistriatus*. Further, *M. meraculus* (Whitley 1948) was originally described as *Histiogamphelus meraculus* and the placement as sister to *H. cristatus* indicates that this generic affiliation may be more appropriate.

H) *Nerophis* Rafinesque 1810 included the monotypic *Entelurus* Dumeril 1870. The type species *N. ophidion* (Linnaeus 1758), is the sister group to *E. aequoreus* and *N. lumbriciformes*. Both an inclusion of *E. aequoreus* in *Nerophis*, or an extension of *Entelurus* to encompass *N. lumbriciformes* are possible. Including *N. maculatus* may help facilitate the decision.

I) *Solegnathus* Swainson 1839 is monophyletic only when including the seadragons *Phycodurus* Gill 1896 and *Phyllopteryx* Swainson 1839, as has before been shown based on a different taxon set [11]. The type species *S. hardwickii* (Gray 1830) groups with *S. lettiensis*, *S. dunckeri* and *S. guentheri* (the latter not sampled here but in [11]). The genus name can stay with this clade. The second clade comprises the seadragons and *Solegnathus spinosissimus* and possibly *S. robustus* [11]. The latter assignment was tentative as *Solegnathus* cf. *robustus* and was separated by short branch lengths from *S. spinosissimus* [11]. Nonetheless, a new genus name for at least *S. spinosissimus* is necessary and an available name is *Castelnauina* Fowler, 1908.

(4) Extensions of known ranges or confirmed wide distributions

Cases where our sampling extended known biogeographic ranges or confirmed wide ranges, in alphabetic order.

Choeroichthys suillus has only been recorded from northern Australia from Perth across north Australia to southern Queensland [43] but the phylogeny here shows a specimen from Palau being sister to a specimen from Queensland.

Corythoichthys polynotatus inhabits coastal waters in the Philippines, Indonesia, and Palau [43] but our phylogeny shows the species extends also to Guam.

Cosmocampus banneri has an usually wide range across much of the Pacific [43], which we confirm with samples from Japan close to the type locality, from the Red Sea and the Philippines.

Dunckerocampus baldwini was treated as a Hawaiian endemic [43], but has also been recorded in West Papua, Indonesia [40]. We include specimens from both Hawaii and Indonesia, confirming this broad range at least in parts of the Central Pacific and the Central Indopacific.

Dunckerocampus boylei is currently known from the Red Sea, Mauritius, and Bali and West Papua, Indonesia [40]. Our sampling extends the range from South Africa to the Philippines.

Dunckerocampus pessuliferus inhabits waters around the Coral Triangle including the Philippines, Indonesia, and northwestern Australia at depths of 15-35 m [43]. Our sampling extends the range to Japan.

Phoxocampus diacanthus is recorded from the Central Indopacific and the eastern Indian Ocean [43]. We include specimens from the Philippines but also Japan, which extends the known range north.

Solegnathus hardwickii is recorded from waters in Japan, the South China Sea to New South Wales and Western Australia [43]. While intermediate occurence in the tropical Central Indopacific are likely, they have not been recorded. Our individual from Malaysia records the species in the tropical Central Indopacific.

Solegnathus paegnius was originally described from Japan. Our specimen from the Philippines extends the known range to the tropical Central Indopacific.

Likely misidentified specimens of Syngnathidae from a previous study [9]**:**

Choeroichthys sculptus (specimen CEO3, SRR5439674, FMNH 124392) is more likely *Choeroichthys suillus*. The specimen is retrieved as the sister group to *C. suillus* from Palau. A photograph of the specimen shows no keeled scutella, which is the defining feature of *C. sculptus*.

Corythoichthys flavofasciatus (specimen CEO92, SRR5439672, KU 5470) is more likely *Corythoichthys intestinalis.* The specimen is retrieved as the sister group to *C. intestinalis* from a closeby locality in the Northern Mariana Islands.

Festucalex wassi (specimen CEO10, SRR5439650, SJL099) is reidentified as *Cosmocampus maxweberi*. Examination of the photographs of the fresh-caught specimen show a long snout, continuous superior trunk ridges, 15 trunk rings, 31 tail rings.

Hippocampus histrix (specimen CEO118, SRR5439636) is more likely *H. barbouri*. It is in the same clade as *H. barbouri* from the Philippines and Malaysia.

Cosmocampus elucens (specimen CEO60, SRR5439667, ANSP 191949) is reidentified as *Halicampus crinitus* based on photographs and its affiliation with specimens of *H. crinitus* from Mexico and Belize.

Syngnathus exilis (specimen CEO57, SRR5439589, SIO:Marine Vertebrates 02-71-2) is now considered *S. californiensis* [42].

Additional Figures

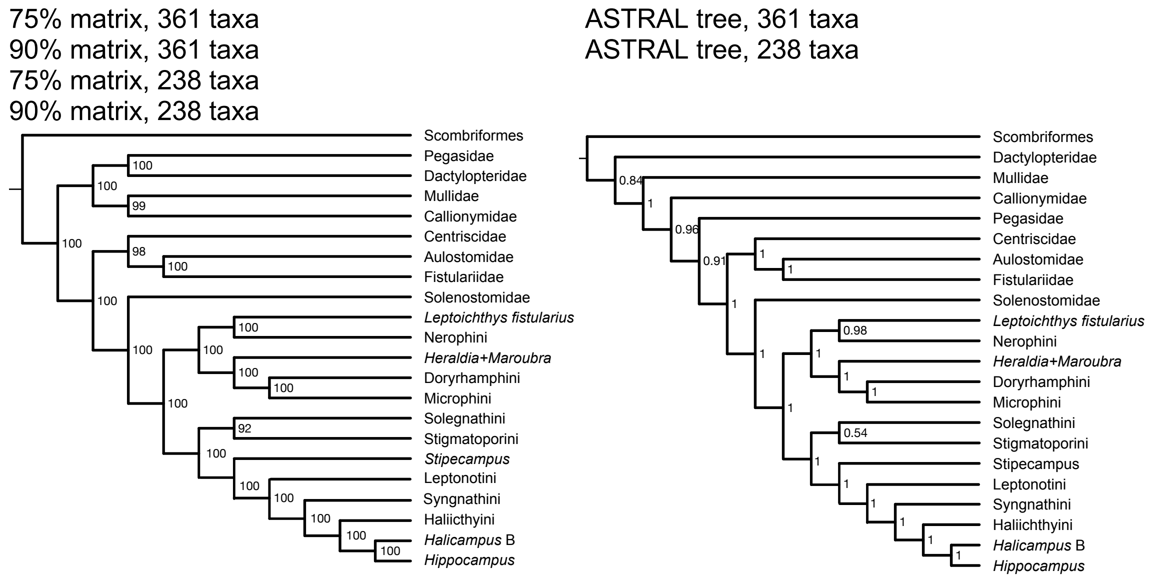

Fig. S1. Simplified topologies of the main lineages of Syngnathiformes comparing concatenation and coalescent-based analyses of the 361-taxa and 268-taxa datasets. Full tree files are shown in the following figures and newick files are available on the FigShare repository. Left: main topology as found in all concatenation analyses, with minor differences in support between matrices. Right: coalescent-based species trees from ASTRAL.

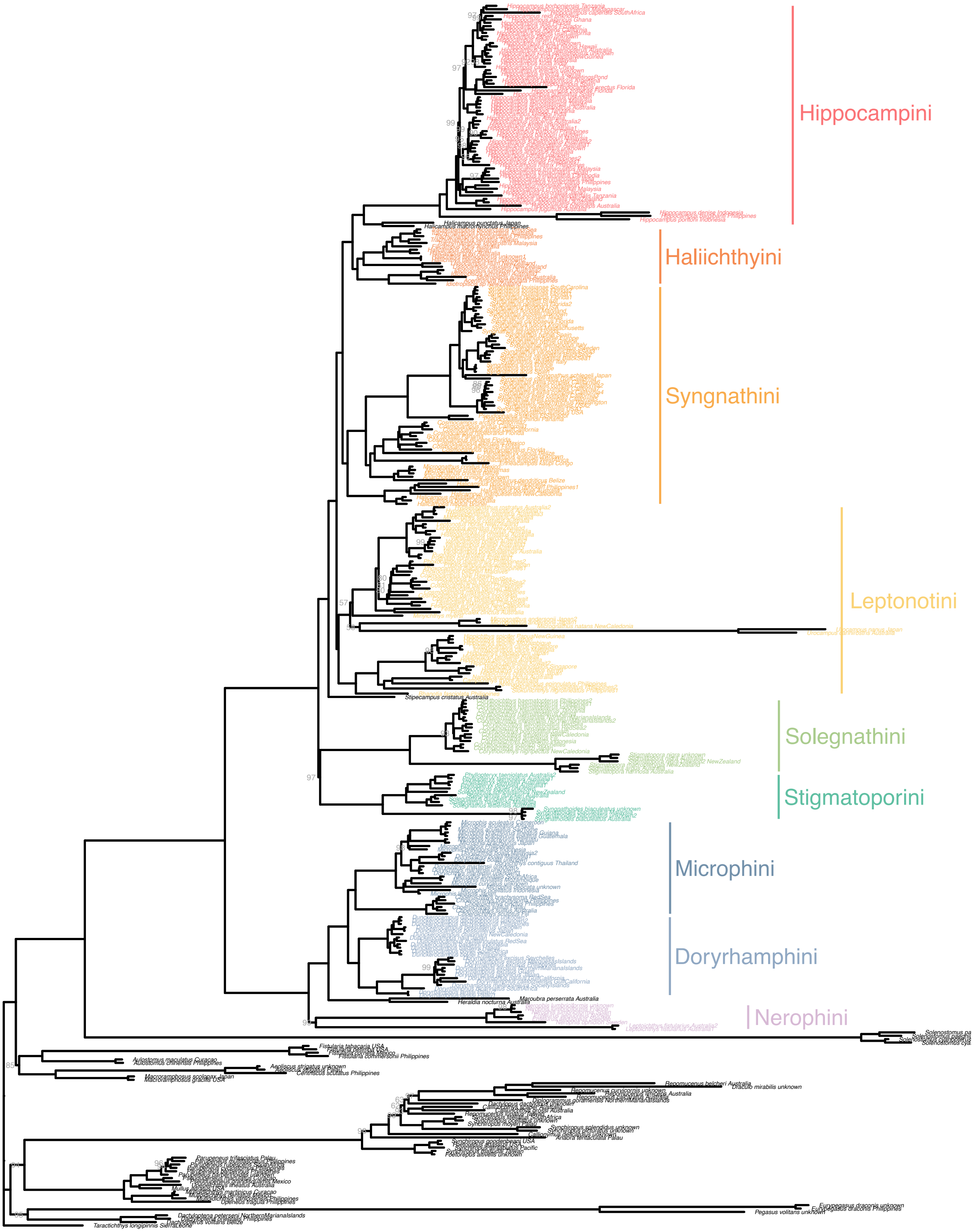

Fig. S2. Maximum likelihood tree of 361 specimens with 90% occupancy. Node labels are bootstrap support values, which are only shown if <100.

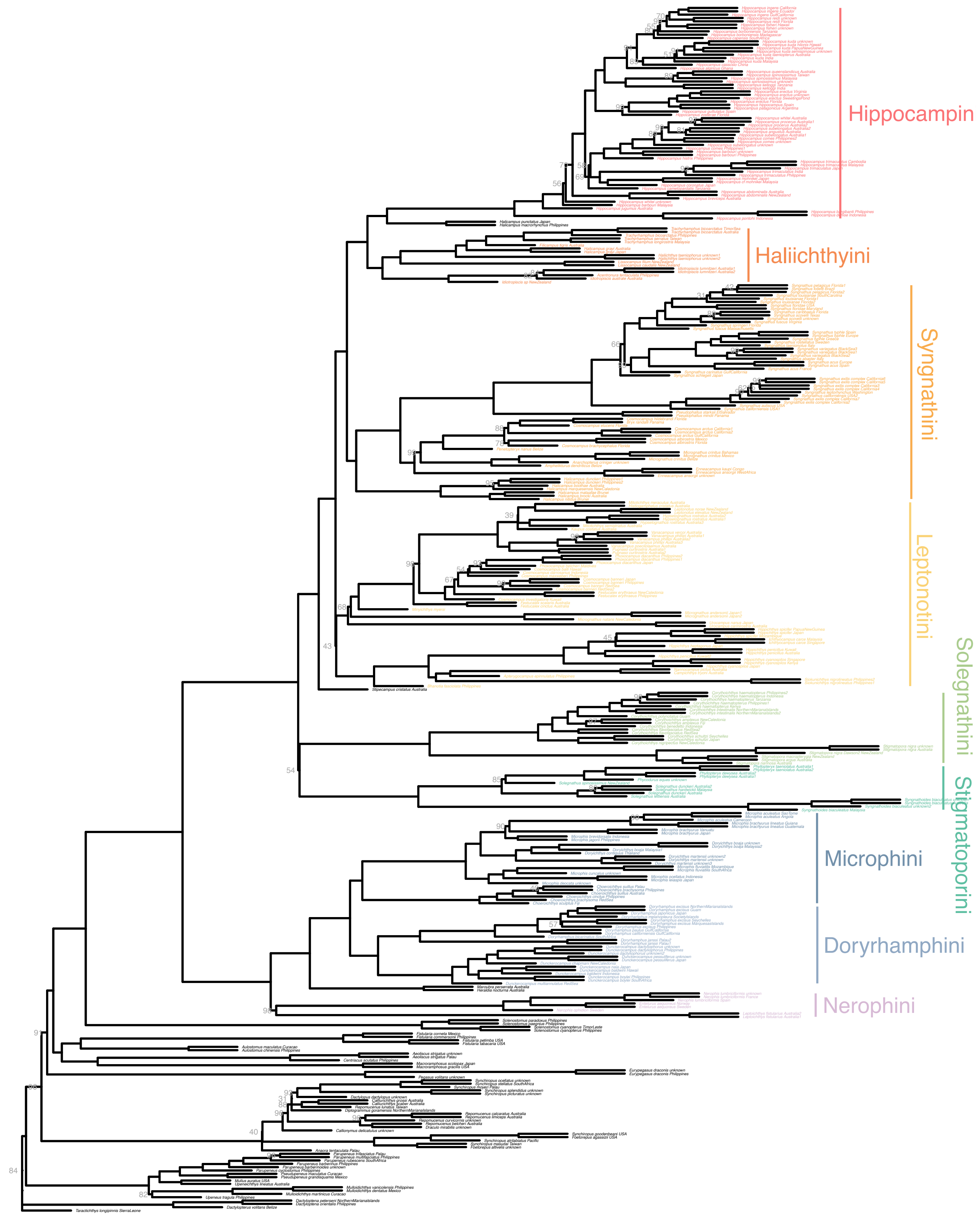

Fig. S3. Coalescent-based species tree from ASTRAL-III summarizing 1309 individual gene trees of 361 taxa. Node labels are posterior probabilities, which are only shown if below 1.00.

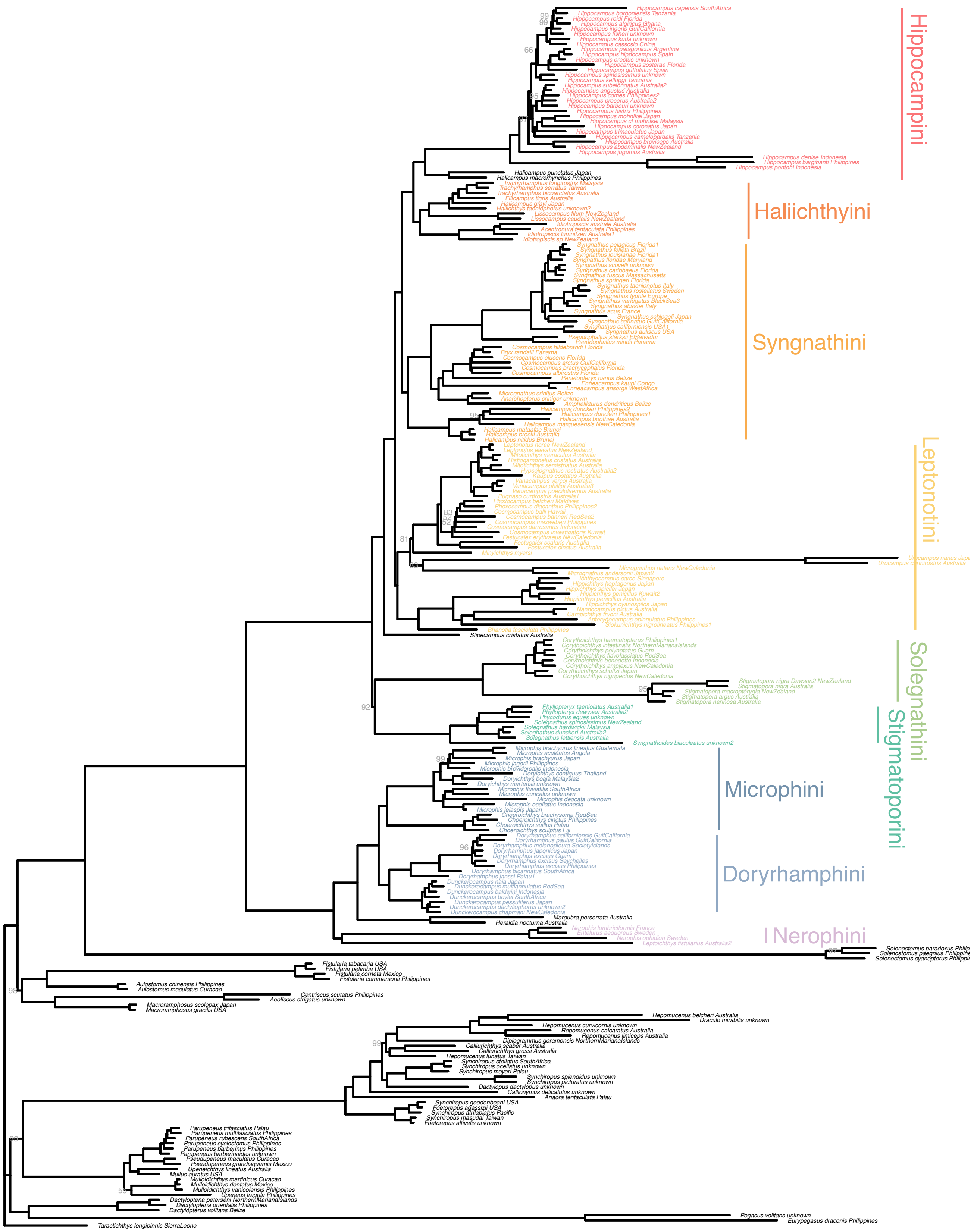

Fig. S4. Maximum likelihood tree of 238 specimens with 75% occupancy. Node labels are bootstrap support values, which are only shown if <100.

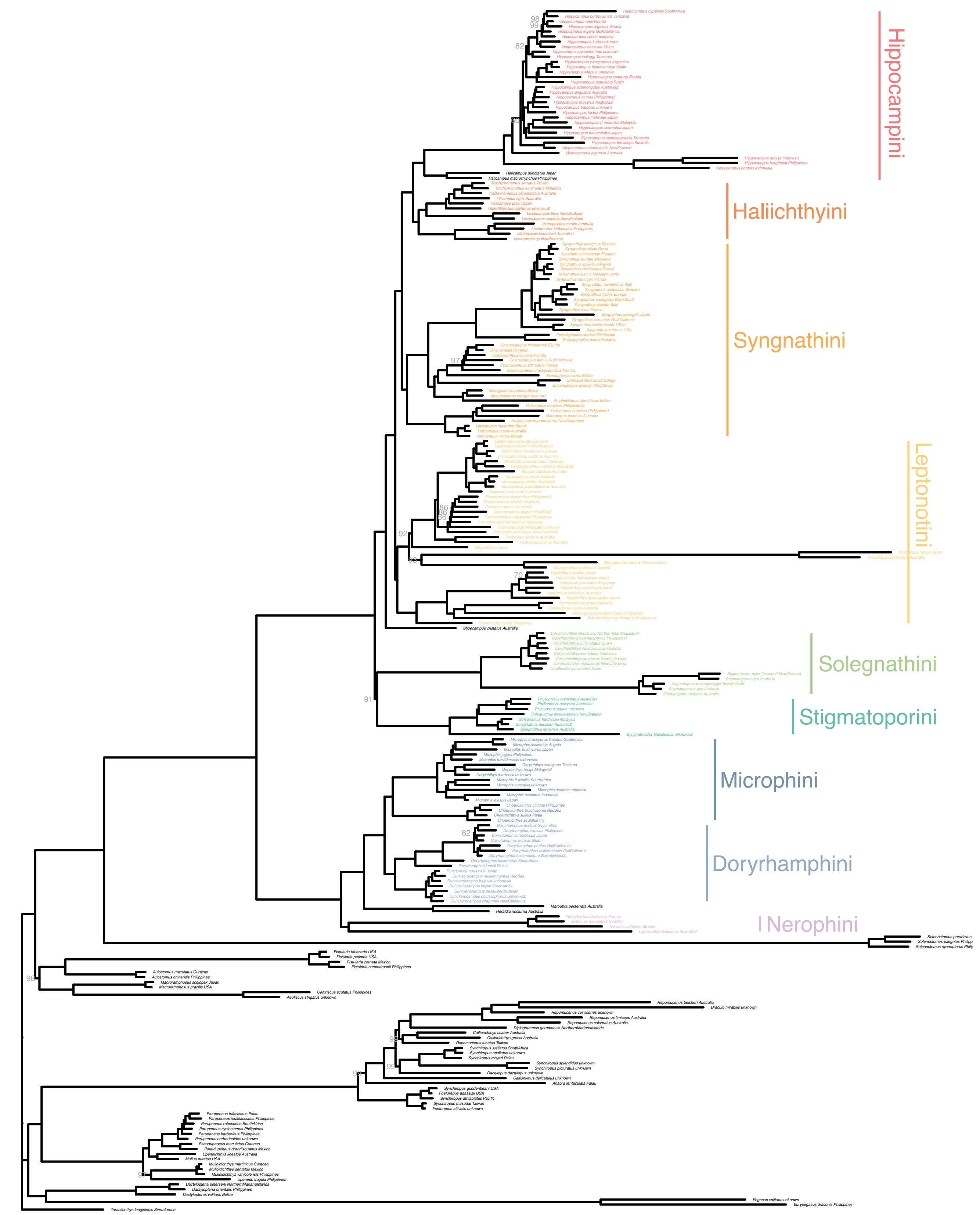

Fig. S5. Maximum likelihood tree of 238 specimens with 90% occupancy. Node labels are bootstrap support values, which are only shown if <100.

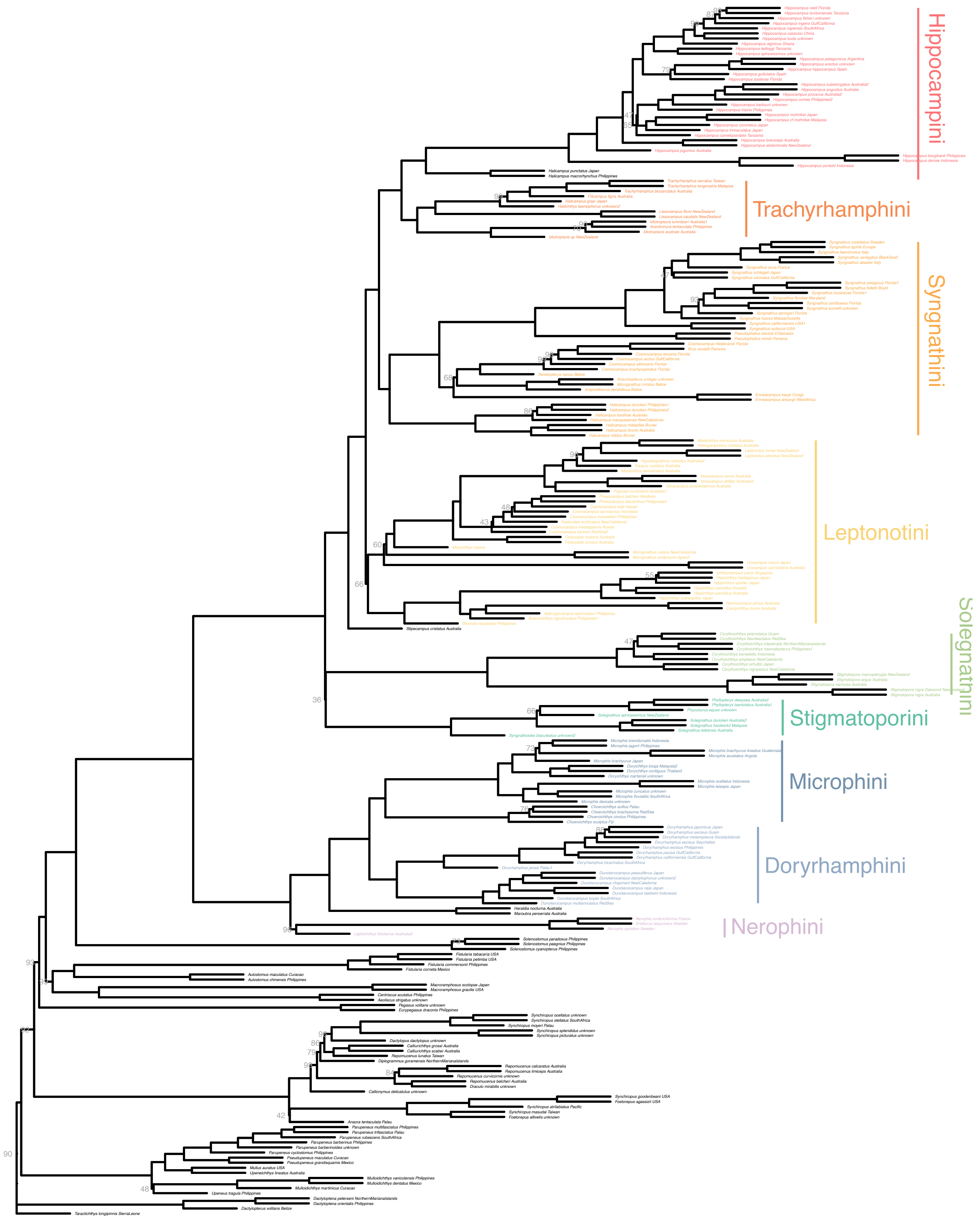

Fig. S6. Coalescent-based species tree from ASTRAL-III summarizing individual gene trees of 238 taxa. Node labels are posterior probabilities, which are only shown if below 1.00.

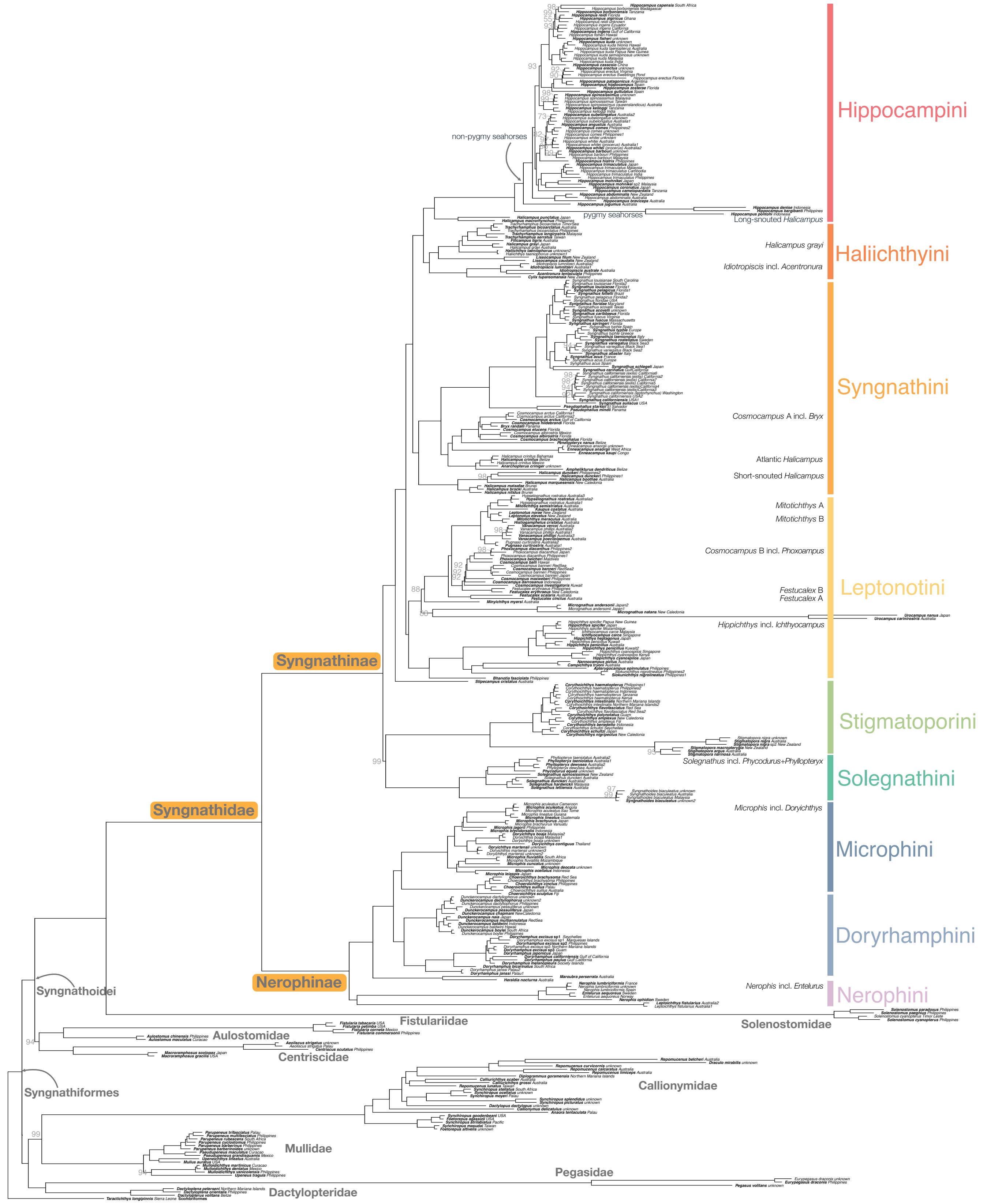

Fig. S7. Complete phylogenetic hypothesis of Syngnathiformes as shown Fig. 1 with all tip labels. Annotations on the right are tribes. Node labels indicate bootstrap support <100.

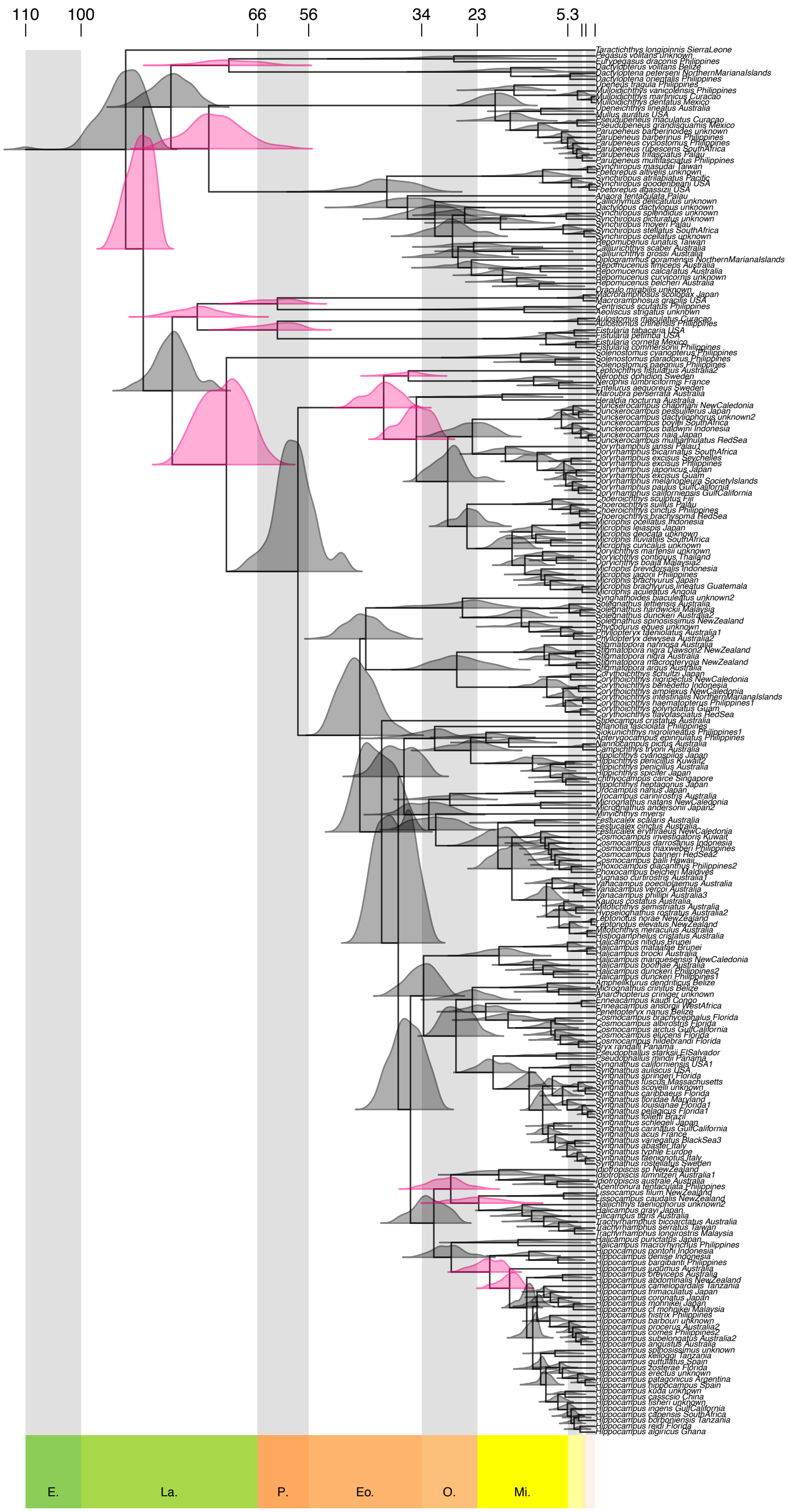

Fig. S8. Calibrated phylogeny from BEAST2 with node age densities. The nodes highlighted in pink are locations of the fossils applied as calibrations. Node age densities were calculated over 100 post-burnin posterior trees from BEAST2.

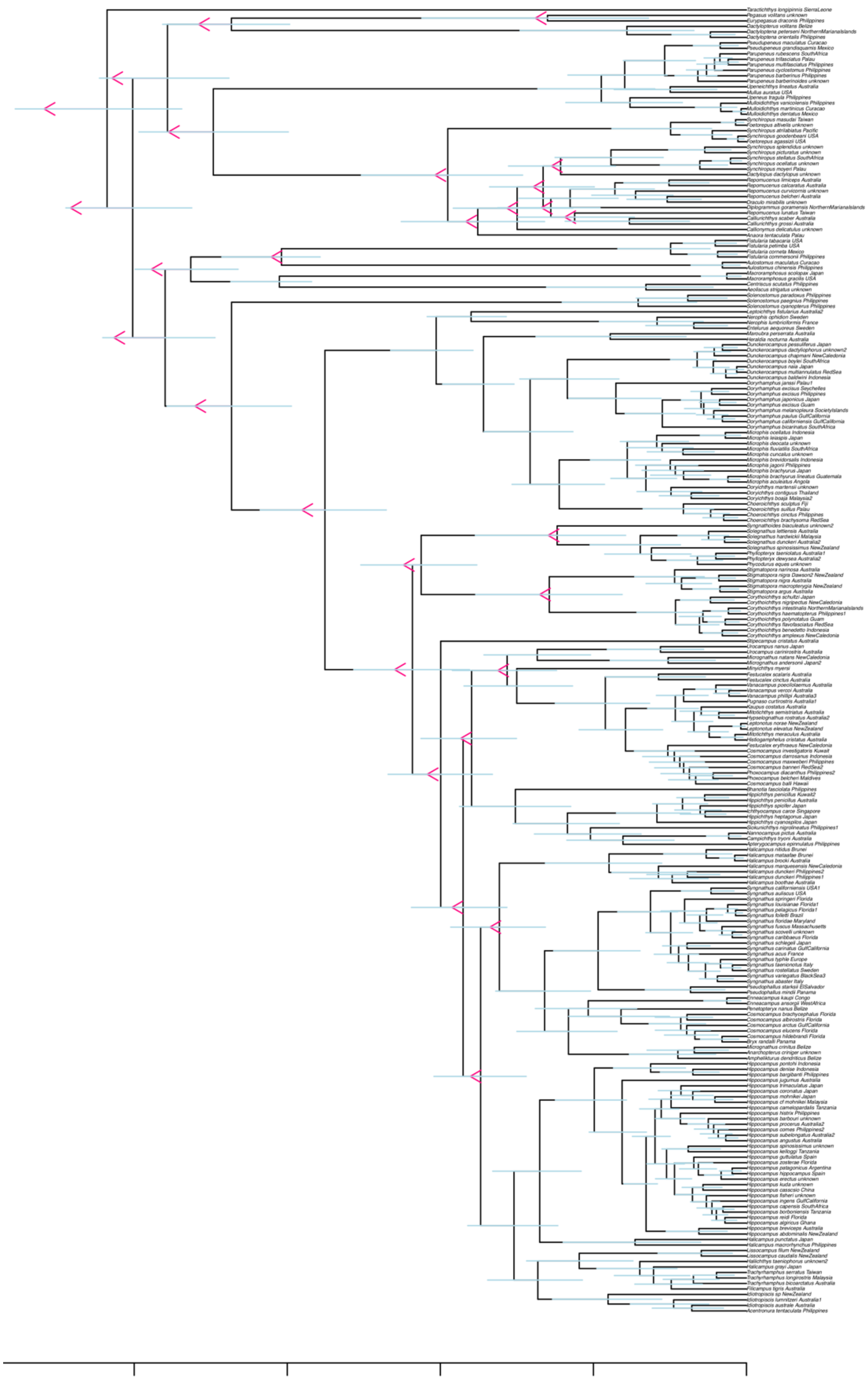

Fig. S9. Calibrated phylogeny from BEAST2 with the fossil calibration set excluding the constraint on Node 1. The arrows indicate the shift of node estimates compared to the full fossil calibration set, if the age difference of a node is more than 1 Ma. Across all nodes, the nodes are on average very slightly older (mean=0.63, median=0.28). The maximum difference between nodes of the two trees is the root node, which is 8.2 Ma older in this analysis.

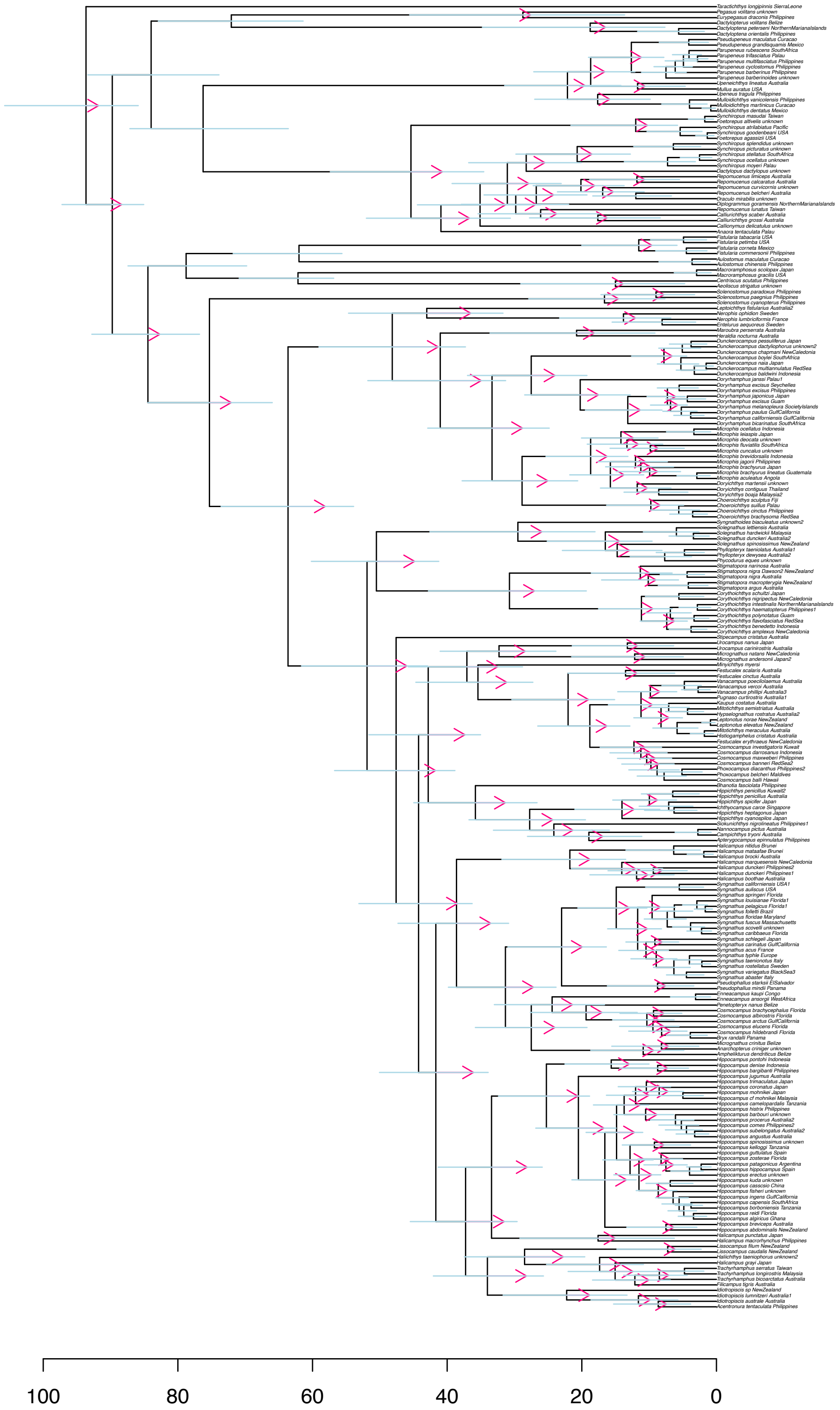

Fig. S10. Calibrated phylogeny from BEAST2 with the fossil calibration set excluding the constraint on any syngnathid nodes. The arrows indicate the shift of node estimates compared to the full fossil calibration set, if the age difference of a node is more than 1 Ma. Across all nodes, the nodes are on average somewhat younger (mean=-1.75, median=-1.21). The maximum difference between nodes of the two trees is -6.8 Ma younger in this analysis.

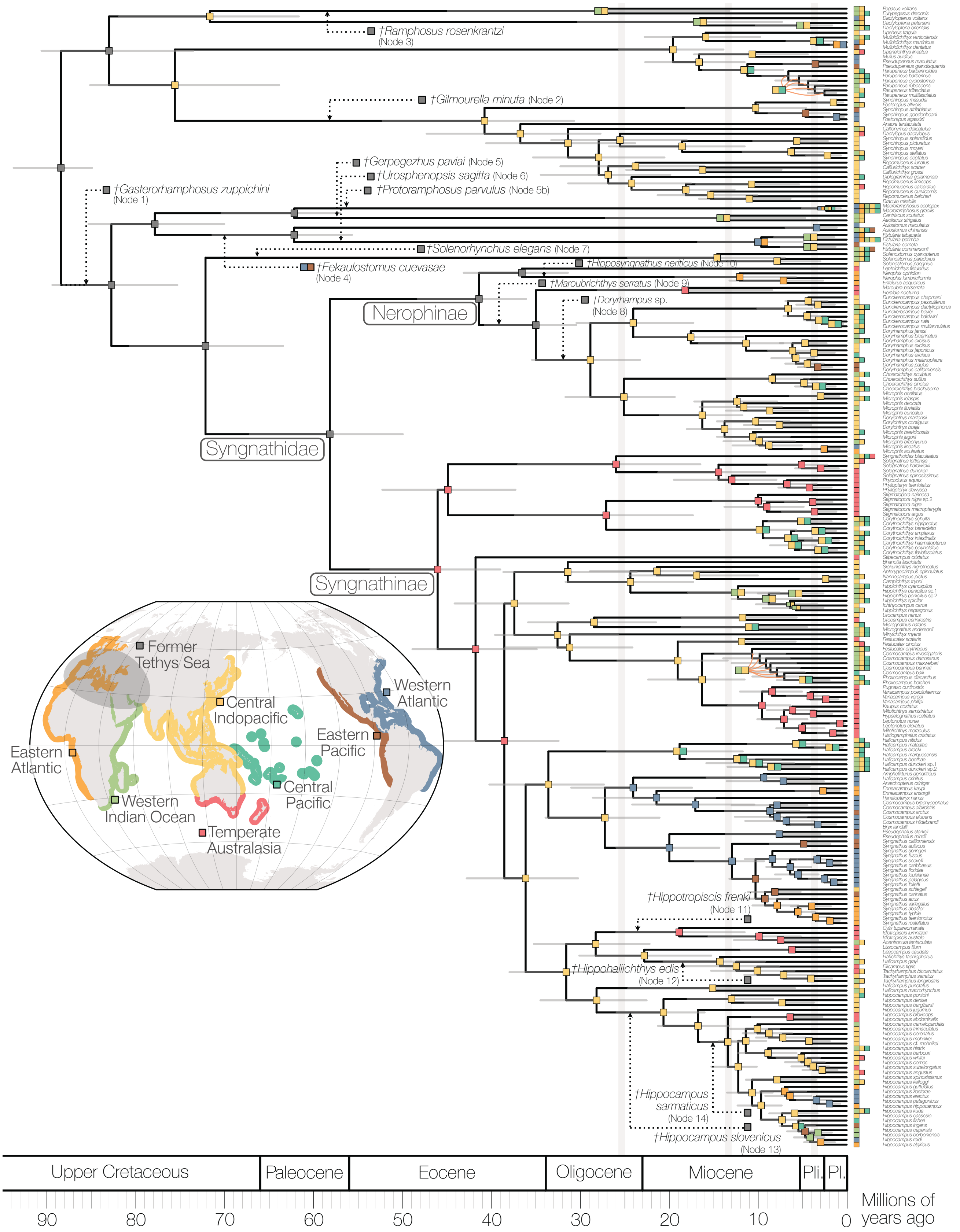

Fig. S11. Dated phylogenetic hypothesis as shown Fig. 5 but with all tip labels.

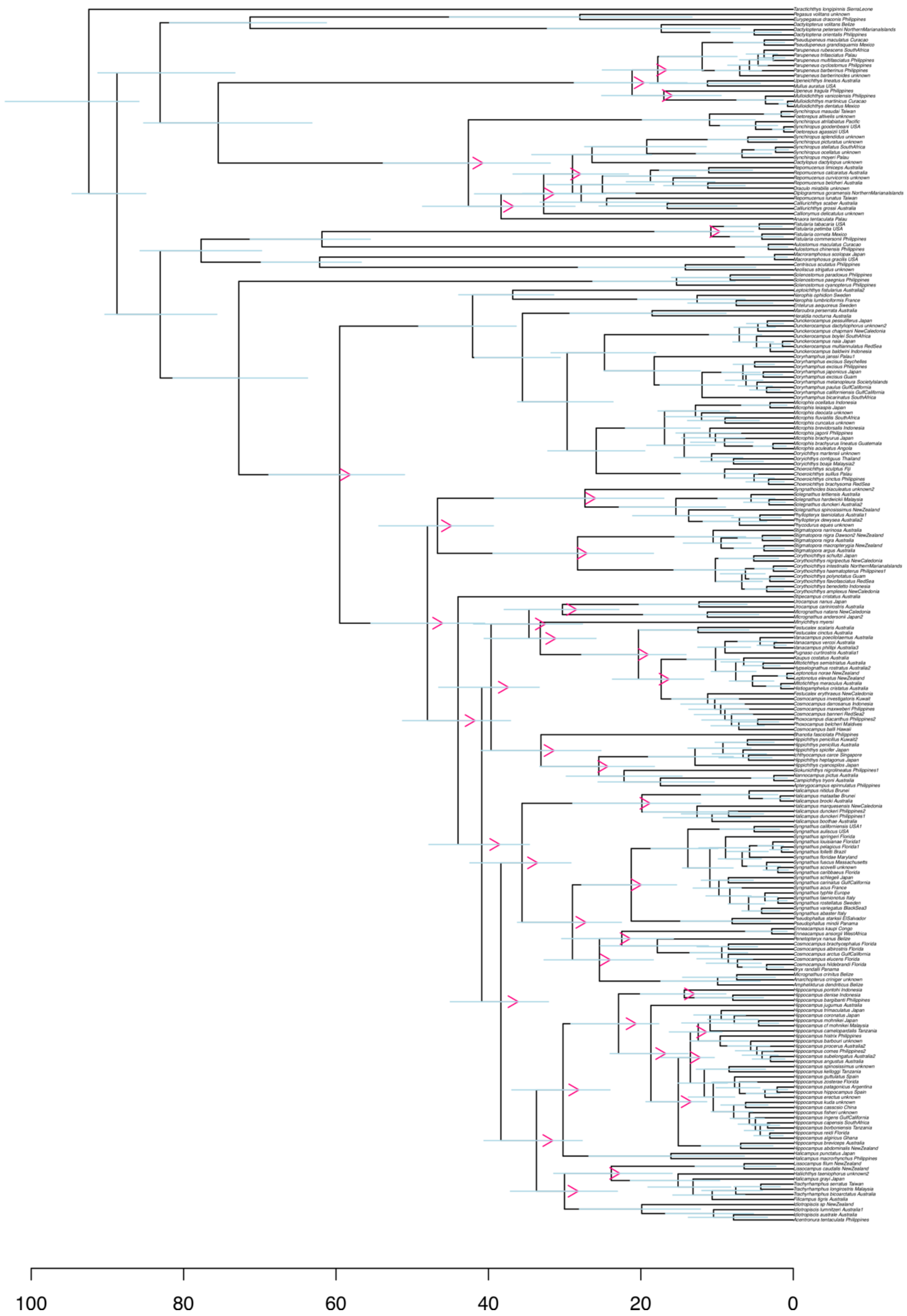

Fig. S12. Calibrated phylogeny from BEAST2 with the fossil calibration set excluding the constraint on the nodes within seahorses (*Hippocampus*). The arrows indicate the shift of node estimates compared to the full fossil calibration set, if the age difference of a node is more than 1 Ma. Across all nodes, the nodes are on average very slightly younger (mean=-0.63, median=-0.47). The maximum difference between nodes of the two trees is on the most recent common ancestor of seahorses, which is -2.3 Ma younger in this analysis.

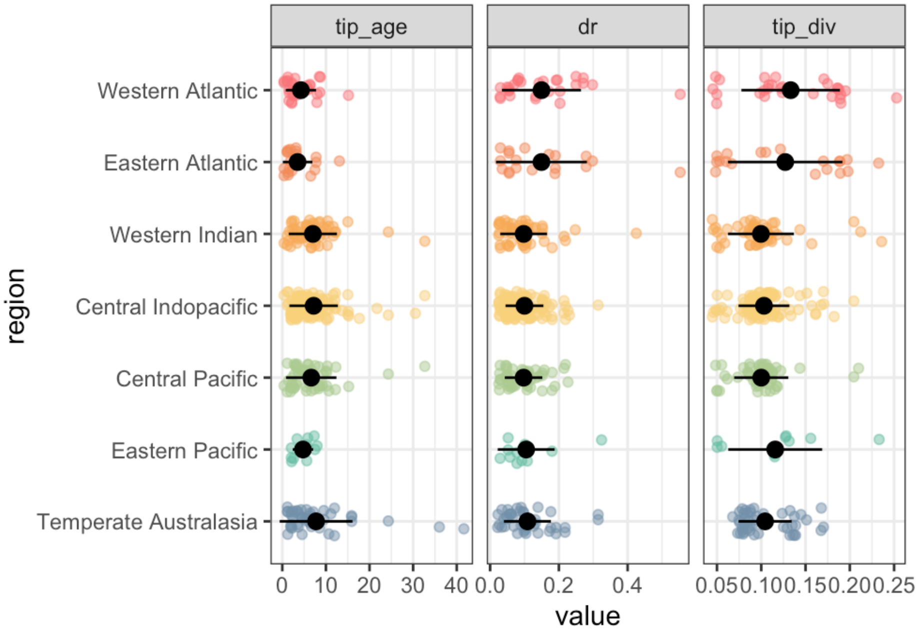

Fig. S13. Estimates of tip age, tip speciation (DR metric) and tip diversification rates for each geographic region. The western and eastern Atlantic region have younger lineages with higher tip diversification rates.

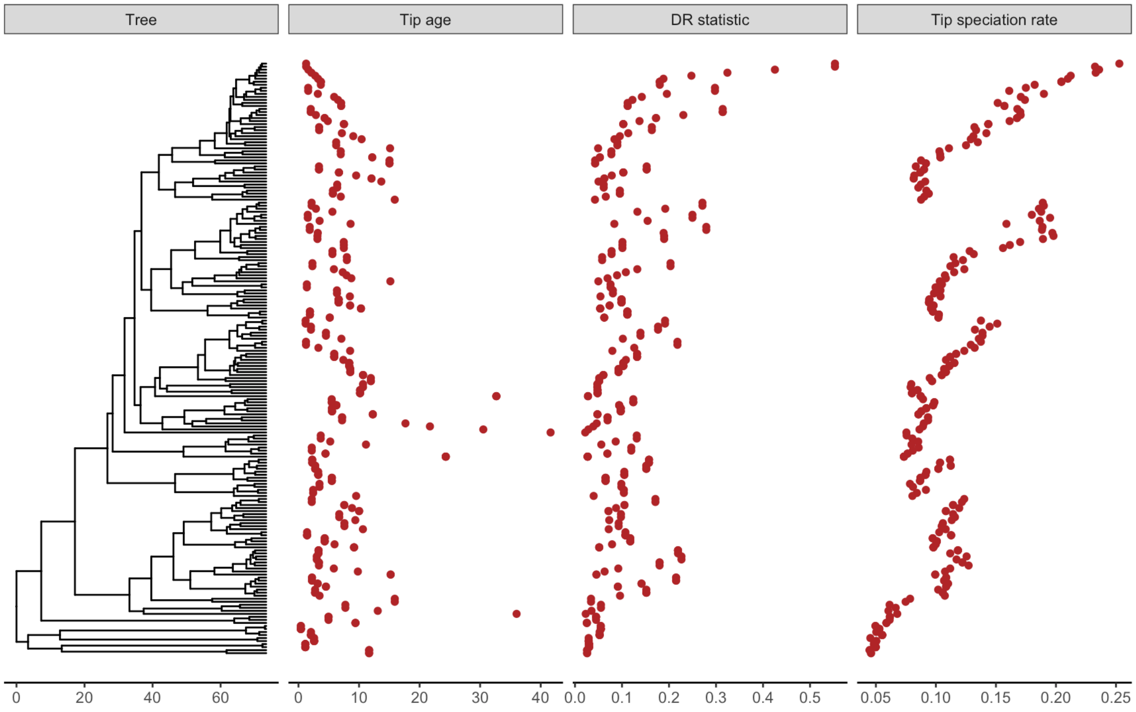

Fig. S14. Distribution of tip ages, DR statistic, and tip speciation rate from ClaDS for every tip of Syngnathoidei.

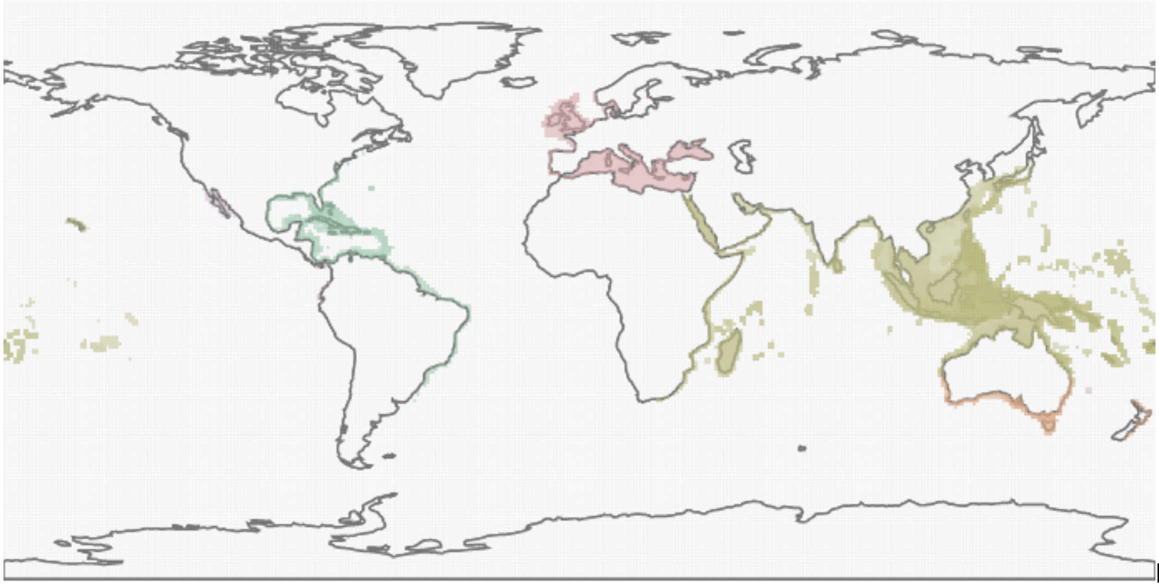

Fig. S15. Delimitation of biogeographic realms using occurrence records for syngnathid and solenostomid species using [123]. The delimitation of regions differs from the main delimitation in grouping the Indian Ocean, Central Indopacific and Central Pacific into one realm.

Additional Tables

Table S1. Number of described and sampled species per genus for Syngnathidae. The sister group Solenostomidae is also included because this study sampled several species. If a genus was paraphyletic, missing species were assigned based on taxonomic characters. Where the species count deviates from published references, see Notes at the bottom of the table for justifications. Most concern the synonymy of species, which largely follows [40, 43, 51, 116]. Cases in which our data shows deviations are annotated in Notes.

Genus or clade	Number species	Number sampled	Proportion sampled	Missing species	Notes
**TOTAL Syngnathidae**	**296**	**183**	**0.62**		
**TOTAL Syngnathidae+ Solenostomidae**	**302**	**186**	**0.62**		
Amphelikturus	1	1	1.00		1
Anarchopterus	2	1	0.50	*Anarchopterus tectus*	
Apterygocampus	1	1	1.00		
Bhanotia	3	1	0.33	*Bhanotia nuda; B. pauciradiata*	
Bryx (see *Cosmocampus* A)					
Campichthys	4	1	0.25	*Campichtys galei; C. nanus; C. tricarinatus*	
Choeroichthys	6	4	0.67	*Choeroichthys latispinosus; C. smithi*	2
Corythoichthys	11	8	0.73	*Corythoichthys insularis; C. ocellatus; C. paxtoni*	3
Cosmocampus Atlantic (Atlantic + Eastern Pacific) incl. *Bryx*	10	6	0.60	*Bryx analicarens; B. dunckeri; B. veleronis; Cosmocampus profundus*	4

Cosmocampus Pacific incl. *Phoxocampus*	9	7	0.78	*Cosmocampus howensis; Phoxocampus tetrophthalmus*	5
Cylix	1	1	1.00		
Doryichthys (see *Microphis*)					
Doryrhamphus	11	9	0.82	*Doryrhamphus aurolineatus; D. negrosensis*	6
Dunckerocampus	7	7	1.00		
Enneacampus	2	2	1.00		
Entelurus (see *Nerophis*)					
Festucalex A *(F. cinctus + F. scalaris)*	5	2	0.40	*Festucalex armillatus; F. gibbsi; F. kulbickii; F. prolixus; F. rufus; F. wassi*	7
Festucalex B (F. *erythraeus*)	4	1	0.25	Same as *Festucalex* A	
Filicampus	1	1	1.00		
Halicampus (short-snouted)	10	7	0.70	*Halicampus edmondsoni; H. spinirostris; H. zavorensis*	8
Halicampus (long-snouted)	2	2	1.00		
Halicampus (Atlantic)	2	1	0.50	*Halicampus erugatus*	13
Halicampus grayi	1	1	1.00		
Haliichthys	1	1	1.00		
Heraldia	1	1	1.00		
Hippichthys incl. *Ichthyocampus*	8	6	0.75	*Hippichthys albomaculosus; H. parvicarinatus*	
Hippocampus (non-pygmy seahorses)	38	29	0.76	*Hippocampus dahli; H. debelius; H. jayakari; H. minotaur; H. paradoxus; H. planifrons; H. pusillus; H. sindonis; H. tyro; H. zebra*	9

Hippocampus (pygmy seahorses)	8	3	0.38	*Hippocampus colemani; H. japapigus; H. nalu; H. satomiae; H. waleananus*	
Histiogamphelus incl. *Mitotichthys meraculus*	3	2	0.67	*Histiogamphelus briggsii*	10
Hypselognathus	2	1	0.50	*Hypselognathus horridus*	
Ichthyocampus (see *Hippichthys*)					
Idiotropiscis incl. *Acentronura*	5	3	0.60	*Acentronura gracilissima; Idiotropiscis larsonae*	11
Kaupus	1	1	1.00		
Leptoichthys	1	1	1.00		
Leptonotus	4	2	0.50	*Leptonotus blainvilleanus; L. vincentae*	
Lissocampus	5	2	0.40	*Lissocampus bannwarthi; L. fatiloquus; L. runa*	
Maroubra	2	1	0.50	*Maroubra yasudai*	
Micrognathus	5	2	0.40	*Micrognathus brevicorpus; M. brevirostris; M. micronotopterus*	12
Microphis incl. *Doryichthys*	26	13	0.50	*Doryichthys deokhatoides; D. heterosoma; Microphis argulus; M. caudocarinatus; M. cruentus; M. dunckeri; M. insularis; M. manadensis; M. mento; M. millepunctatus; M. pleurostictus; M. retzii; M. spinachioides*	14
Minyichthys	4	1	0.25	*Minyichthys brachyrhinus; M. inusitatus; M. sentus*	
Mitotichthys A	3	1	0.33	*Mitotichthys mollisoni, M. tuckeri*	10
Mitotichthys B (see *Histiogamphelus*)					
Nannocampus	4	1	0.25	*Nannocampus elegans; N. subosseus; N. weberi*	
Nerophis incl. *Entelurus*	4	3	0.75	*Nerophis maculatus*	

Penetopteryx	2	1	0.50	*Penetopteryx taeniocephalus*	
Phoxocampus (see *Cosmocampus*)					
Phycodurus	1	1	1.00		
Phyllopteryx	2	2	1.00		
Pseudophallus	4	2	0.50	*Pseudophallus brasiliensis; P. elcapitanensis*	
Pugnaso	1	1	1.00		
Siokunichthys	5	1	0.20	*Siokunichthys bentuviai; S. breviceps; S. herrei; S. southwelli;*	
Solegnathus	3	3	1.00		
Solegnathus spinosissimus	2	1	0.50	*Solegnathus robustus*	
Solenostomus	6	3	0.50	*Solenostomus armatus; S. halimeda; S. leptosoma*	16
Stigmatopora	7	5	0.71	*Stigmatopora harastii; S. nigra* lineage 2 sensu [30]	17
Stipecampus	1	1	1.00		
Syngnathoides	1	1	1.00		
Syngnathus	33	18	0.55	*Syngnathus affinis; S. caspius; S. chihiroe; S. dawsoni; S. euchrous; S. insulae; S. macrobrachium; S. macrophthalmus; S. makaxi; S. phlegon; S. safina; S. schmidti; S. temminckii; S. tenuirostris; S. watermeyeri*	
Trachyrhamphus	3	3	1.00		
Urocampus	2	2	1.00		
Vanacampus	4	3	0.75	*Vanacampus margaritifer*	

1. [116] notes that additional species may exist in the eastern Atlantic, and two or more in the Pacific but does not provide sufficient information to assess.
2. [40] lists *Choeroichthys valencienni* as valid but we follow [43] in considering it as synonym of *C. brachysoma*.

3. [40] lists *Corythoichthys conspillatus* as valid but we follow [43] in considering it a synonym of *C. flavofasciatus*. [40] lists *C. isigakius* as valid but we follow [12] in considering it a synonym of *C. haematopterus*. [40] lists *C. waitei* as valid but we follow [43] in considering it a synonym of *C. intestinalis*. We recognize the 10 species that were reviewed by [43], in addition to the more recently described *C. benedetto*, to a total of 11 species.
4. *Cosmocampus* clade A is largely distributed in the Atlantic Ocean with the exception of one group in the tropical and temperate eastern Pacific (*C. arctus* complex). Our current sampling cannot assess the status of the subspecies of *C. arctus* (*C. a. heraldi, C. a. coccineus*. Until further evidence is available, we treat them as members of *C. arctus*. Further, *Bryx randalli* is nested within the Atlantic *Cosmocampus* group and is included in this count.
5. *Cosmocampus* clade B is distributed in the Pacific Ocean and also contains species of *Phoxocampus*. We assume that the Pacific species that we have not sampled (*C. howensis, P. tetrophthalmus*) group with *Cosmocampus* B. [40] lists *P. kampeni* as a valid species but we follow [43] in considering it a synonym of *P. tetrophthalmus*.
6. Two missing species are 1) *Doryrhamphus negrosensis* with a subspecies *D. n. malus* that we consider as a member of the same species until data is available to assess; 2) *D. aurolineatus* was described later and is considered valid. [40] treats *D. extensus* as a valid species but we follow [43] in the synonymization with *D. excisus* until samples from the type locality of *D. extensus* (Japan) are available. *Doryrhamphus excisus* specimens appear several times in the phylogeny and will need further investigation. In the Mexican eastern Pacific, we treat *D. californiensis* and *D. paulus* as species given their long branches in the phylogeny. This interpretation differs from [40, 43], which consider *D. paulus* and *D. californiensis* subspecies of *D. excisus*. In the Indopacific, "*Doryrhamphus excisus*" forms at least 3 clades that are separated by long branches: 1) One species is found in Guam and Mariana Islands, 2) one in the Seychelles and Marquesas, 3) one in the Philippines. The assignment to the subspecies recognized by [43] is challenging because the type locality of *D. e. excisus* is unknown and we are missing a specimen from the type locality (Red Sea) of *D. e. abbreviatus.*
7. *Festucalex* (9 species total) is paraphyletic. The 6 unsampled species are more recent descriptions than [43] and are considered valid. No previous information exists to allow us to form a hypothesis about their phylogenetic position in either clade. We therefore arbitrarily split the unsampled species onto each clade, so that 3 species are missing in each. *Festucalex amakusensis* and *F. townsendi* are listed by [40] but we follow [43] in considering it as a synonym of *F. erythraeus* until further evidence is presented.
8. *Halicampus A* is a clade of short-snouted animals. Two specimens identified as *H. dunckeri* from the Philippines are sister to each other but have long branches separating them and we consider them as tentatively as separate species. Further investigation into this species is needed to evaluate if they correspond to any of the 4 morphotypes of *H. dunckeri* identified by [43]. This brings the number of total species in *Halicampus* A to 10, of which we sampled 7.
9. [51] recognize 41 species of *Hippocampus*, of which 8 are pygmy seahorses and 33 are non-pygmy seahorses. Additional species are *H. casscsio*, which was described after [51] and is shown in [49] and here to be a distinct lineage; *H. mohnikei* consists of at least two species as shown here and suggested in [51] based on barcode data; *H. borboniensis* is treated as a synonym of *H. kuda* [51] but these species are shown here to be in separate clades and *H. borboniensis* is thus considered valid. As [51], we treat *H. angustus* and *H. subelongatus* tentatively as separate species because individuals formed reciprocally monophyletic groups. *Hippocampus procerus* is considered a synonym of *H. whitei* by [51], which we confirm here. This brings the species count for *Hippocampus* (non-pygmy) to 38, of which we sampled 29.
10. We show that *Mitotichthys* is paraphyletic and the species is therefore counted as a member of *Histiogamphelus*.
11. [43] treats *Idiotropiscis* as a subgenus of *Acentronura*, which is shown here to be nested within the sampled members of *Idiotropiscis*. We count all species of [43], and a more recently described species (*Idiotropiscis lumnitzeri*). This adds to a total of 5 species of *Idiotropiscis* incl. *Acentronura*, of which we have sampled 3. We follow [43] in considering *A. mossambica* and *A. breviperula*, which [40] treats as a valid species, as synonyms of *A. tentaculata*.

12. We sampled two species and are missing 3 species, one of which was described after [43] and is added to the count here. [40] lists two subspecies of *M. brevirostris* as separate species, while we follow [43] in treating them as a single species until further evidence is available. This sums to a total number of 5 species of Pacific *Micrognathus* species.
13. We assume that the unsampled Atlantic species *Halicampus erugatus* forms a clade with *H. crinitus* from the Atlantic.
14. *Doryichthys* is contained inside *Microphis* and we therefore sum the number of described species for these two clades. *Doryichthys contiguus* was described after [43] and is added as a valid species. *Microphis* was divided into 5 subgenera [43] but our study does not confirm the monophyly of these and we hence treat them as *Microphis*. *Microphis brachyurus* was divided into 4 subspecies with distinct geographic ranges. We sequenced specimens for 3 of these putative subspecies and found long branches separating them. We therefore treat the subspecies as separate species: *M. brachyurus, M. aculeatus, M. lineatus* and add *M. (brachyurus) millepunctatus* as an unsampled tentative species. [40] lists *Coelonotus biocellatus* and *M. platyrhynchus* as valid species but we follow [43] in synonymizing with *M. argulus*; similarly *M. vaillantii* and *M. yoshi* are synonyms of *M. leiaspis*.
15. For *Mitotichthys*, we assumed that the two unsampled species group with *M. semistriatus*, rather than *M. meraculus* which is part of *Histiogamphelus* until further evidence is available.
16. *Solenostomus paegnius* is considered a junior synonym of *S. cyanopterus* [41] but the branch lengths separating them indicate distinct species.
17. *Stigmatopora nigra* was shown to have strong genetic structure across its range, possibly warranting description of 2 new species in addition to the nominal *S. nigra* [61]. We count these as valid species, which brings the species count to 6. We sampled one of the cryptic lineages of *S. nigra* from New Zealand, in addition to nominal *S. nigra.*

Table S2. Comparison of diagnostic characters between the fossils and extant members of Nerophinae.

	†*Maroubrichthys serratus* Parin, 1992	†*Doryrhamphus* sp. Micklich & Parin, 1996	†*Hipposyngnathus neriticus* Jerzmańska, 1968	*Doryrhampus*	*Dunckerocampus*	*Choeroichthys*	*Microphis* incl. *Doryichthys*	*Heraldia*	*Maroubra*	*Nerophis* incl. *Entelurus*	*Leptoichthys*
Pouch plates	Y	?	Y	N	N	Y	Y	N	N	Y/N (loss)	Y
Opercle ridge	Y	Y	N	Y	Y	Y	Y	Y	Y	N	Y/N
Caudal fin	Y	Y	?*	Y	Y	Y	Y	Y	Y	N	Y
Scutella with keels	N	Y	N	N	N	Y	Y	N	N	N	N
Distal spines on body ridges	Y	Y	N	Y	Y	Y	Y	Y	Y	N	N
Superior ridges continuous	?	?	Y	N	N	Y	N	N	N	Y	N
Inferior ridges continuous	?	?	N	N	N	N	N	Y	Y	N	Y
Lateral ridge curve	?	?	ventrally	ventrally	ventrally	ventrally	ventrally	dorsally	dorsally	ventrally	straight

*Displayed in the reconstruction but not preserved in the specimens?

Table S3. Number of specimens and species sequenced in this study and the three most recent studies of Syngnathidae or Syngnathiformes.

Number	This study - Total	This study - New sequences	Hamilton et al. 2017 [44]	Longo et al. 2017 [9]	Santaquiteria et al. 2021** [11]
Specimens Syngnathiformes + 1 outgroup	361	248	95	113	77 (185)
Species Syngnathiformes + 1 outgroup	238	118	95	109*	62 (170)
Specimens Syngnathidae	303	244	91	59	14 (73)
Species Syngnathidae	183	117	91	57*	14 (71)

*[11] identified 4 specimens from [9] that were likely members of the same species. The number of species count is therefore lower than the number of specimens.

**The study included the data from [9]. Numbers are samples added by the study and in brackets the number of samples including the previous dataset.

Table S4. Statistics for the matrices and loci used in phylogenetic analyses. Individual locus statistics are given in Dataset 2.

	361 taxa			237 taxa		
	All loci	75% matrix	90% matrix	All loci	75% matrix	90% matrix
Number of loci	1309	934	641	1310	934	680
Locus length (sum)	302,199	236,733	171,640	298,969	230,623	174,843
Locus length (mean)	230.5	252.9	266.9	228.9	246.4	256.4
Number of taxa (mean)	261.0	328.0	338.2	175.1	218.3	224.0
% of variable sites (mean)	52.5	58.2	57.4	50.1	55.4	54.7
% gap (mean)	4.1	3.6	3.5	4.0	3.6	3.4

Table S5. Dispersal multipliers between biogeographic regions in three time slices. Dispersal between disconnected areas (require transversal through at least one other area) was modeled with a dispersal multiplier of 0.001, dispersal between distant areas (requiring transoceanic dispersal or dispersal around the Cape of Hope) was modeled with a dispersal multiplier of 0.1, and dispersal between adjacent areas were modeled with a multiplier of 1.0.

3.5-0 Ma (closure of the Great American Seaway to present)

Western Atlantic	Eastern Atlantic	Western Indian	Central Indopacific	Central Pacific	Eastern Pacific	Temperate Australasia	Tethys Sea
1.000	0.100	0.001	0.001	0.001	0.001	0.001	0.001
0.100	1.000	0.100	0.001	0.001	0.001	0.001	0.001
0.001	0.100	1.000	1.000	0.001	0.001	0.100	0.001
0.001	0.001	1.000	1.000	1.000	0.001	1.000	0.001
0.001	0.001	0.001	1.000	1.000	0.100	1.000	0.001
0.001	0.001	0.001	0.001	0.100	1.000	0.001	0.001
0.001	0.001	0.100	1.000	1.000	0.001	1.000	0.001
0.001	0.001	0.001	0.001	0.001	0.001	0.001	0.001

13.8-3.5 Ma (closure of Tethys Sea to closure of the Great American Seaway)

Western Atlantic	Eastern Atlantic	Western Indian	Central Indopacific	Central Pacific	Eastern Pacific	Temperate Australasia	Tethys Sea
1.000	0.100	0.001	0.001	0.001	1.000	0.001	0.001
0.100	1.000	0.100	0.001	0.001	0.001	0.001	0.001
0.001	0.100	1.000	1.000	0.001	0.001	0.100	0.001
0.001	0.001	1.000	1.000	1.000	0.001	1.000	0.001
0.001	0.001	0.001	1.000	1.000	0.100	1.000	0.001
1.000	0.001	0.001	0.001	0.100	1.000	0.001	0.001
0.001	0.001	0.100	1.000	1.000	0.001	1.000	0.001
0.001	0.001	0.001	0.001	0.001	0.001	0.001	0.001

25-13.8 (Indoaustralian Plate meeting Eurasian Plate to closure of the Tethys Sea)

Western Atlantic	Eastern Atlantic	Western Indian	Central Indopacific	Central Pacific	Eastern Pacific	Temperate Australasia	Tethys Sea
1.000	0.100	0.001	0.001	0.001	1.000	0.001	1.000

0.100	1.000	0.100	0.001	0.001	0.001	0.001	1.000
0.001	0.100	1.000	1.000	0.001	0.001	0.100	1.000
0.001	0.001	1.000	1.000	1.000	0.001	1.000	1.000
0.001	0.001	0.001	1.000	1.000	0.100	1.000	0.001
1.000	0.001	0.001	0.001	0.100	1.000	0.001	0.001
0.001	0.001	0.100	1.000	1.000	0.001	1.000	0.001
1.000	1.000	1.000	1.000	0.001	0.001	0.001	1.000

90-25.0 (root to Indo-Australian Plate meeting Eurasian Plate)							
Western Atlantic	Eastern Atlantic	Western Indian	Central Indopacific	Central Pacific	Eastern Pacific	Temperate Australasia	Tethys Sea
1.000	1.000	0.001	0.001	0.001	1.000	0.001	1.000
1.000	1.000	0.100	0.001	0.001	0.001	0.001	1.000
0.001	0.100	1.000	1.000	0.001	0.001	0.100	1.000
0.001	0.001	1.000	1.000	1.000	0.001	0.100	1.000
0.001	0.001	0.001	1.000	1.000	0.100	0.100	0.001
1.000	0.001	0.001	0.001	0.100	1.000	0.001	0.001
0.001	0.001	0.100	0.100	0.100	0.001	1.000	0.001
1.000	1.000	1.000	1.000	0.001	0.001	0.001	1.000

Table S6. Parameters and estimates from BioGeoBEARS analysis. Model fit was compared using log likelihood (lnL) and AIC.

		Free parameters					Model fit	
Models	lnL	N	d	e	j	w	AIC	AIC weight
DEC	-824.4	2	0.030	0.014	N/A	N/A	1653.0	1.10E-59
DEC+j	-781.4	3	0.020	0.002	0.095	N/A	1569.0	2.10E-41
DEC+w	-782.4	3	0.021	0.011	N/A	0.160	1571.0	7.80E-42
DEC+j+w	-714.6	4	0.013	0.000	0.041	0.130	1437.0	7.50E-13
DIVALIKE	-831.5	2	0.032	0.011	N/A	N/A	1667.0	9.40E-63
DIVALIKE+j	-794.1	3	0.023	0.003	0.069	N/A	1594.0	5.90E-47
DIVALIKE+w	-788.4	3	0.022	0.009	N/A	0.150	1583.0	1.80E-44
DIVALIKE+j+w	-725.3	4	0.016	0.000	0.033	0.160	1459.0	1.60E-17
BAYAREALIKE	-875.1	2	0.030	0.043	N/A	N/A	1754.0	1.10E-81
BAYAREALIKE+j	-742.3	3	0.013	0.004	0.110	N/A	1491.0	1.80E-24
BAYAREALIKE+w	-818.3	3	0.016	0.036	N/A	0.075	1643.0	1.90E-57
**BAYAREALIKE+j+w**	**-686.7**	**4**	**0.008**	**0.002**	**0.036**	**0.048**	**1381.0**	**1.000**
